# Long-Term Euxinia Restricts Microbial Methane Removal in Eutrophic Coastal Basins

**DOI:** 10.1021/acs.est.5c05066

**Published:** 2025-10-08

**Authors:** Jessica Venetz, Nicky Dotsios, Olga M. Żygadłowska, Wytze K. Lenstra, Niels A.G.M van Helmond, Christoph Humborg, Katherine D. McMahon, Dina in ’t Zandt, Caroline P. Slomp, Mike S. M. Jetten, Annelies J. Veraart

**Affiliations:** † Department of Microbiology, Radboud Institute for Biological and Environmental Sciences, 6029 Radboud University, 6500 HC Nijmegen, The Netherlands; ‡ Department of Earth Sciences, Faculty of Geosciences, 84481 Utrecht University, 3584 CB Utrecht, The Netherlands; § Baltic Sea Centre, 7675 Stockholm University, SE 106 91 Stockholm, Sweden; ∥ Departments of Civil and Environmental Engineering and Bacteriology, University of Wisconsin-Madison, Madison, Wisconsin 53706-1314, United States; ⊥ Department of Ecology, Radboud Institute for Biological and Environmental Sciences, Radboud University, 6500 HC Nijmegen, The Netherlands; # Terrestrial Ecology, Netherlands Institute of Ecology, 6700 HB Wageningen, The Netherlands

**Keywords:** tipping-point, long-term stratification, methanotrophic bacteria, anoxia, microbial community

## Abstract

In eutrophic coastal waters, aerobic methane-oxidizing bacteria (MOB) mitigate methane emissions by oxidizing benthic methane even in the stratified, anoxic water column. However, ongoing warming and eutrophication lead to extended stratification periods, enhancing anoxic and sulfidic conditions (euxinia), potentially affecting methane removal capacity. Here we compared overall water column methane removal between sites with irregular, seasonal and longer-term euxinia in the Stockholm Archipelago during summer 2022. The highest water–air methane emissions, bottom water–methane and sulfide accumulation, and the lowest methane oxidation potential were observed under longer-term euxinic bottom water conditions. While MOB relative abundance and potential activity indicated high functioning of the methane biofilter in the seasonally euxinic bottom water layer, the methane-filtering potential was much lower in the longer-term euxinic bottom water. Under persistent euxinic conditions, overall bacterial diversity and microbial network connectivity were lower, likely following a simultaneous shift in redox conditions and a shift toward anaerobic sulfur-cycling. This shift may force MOB to retreat from the euxinic bottom water into the narrow oxycline, reducing the capacity of the methane biofilter and resulting in higher methane emissions. These findings highlight the positive feedback loop that can further amplify oceanic methane emissions, particularly from eutrophic and shallow coastal waters prone to prolonged stratification under global warming.

## Graphical abstract

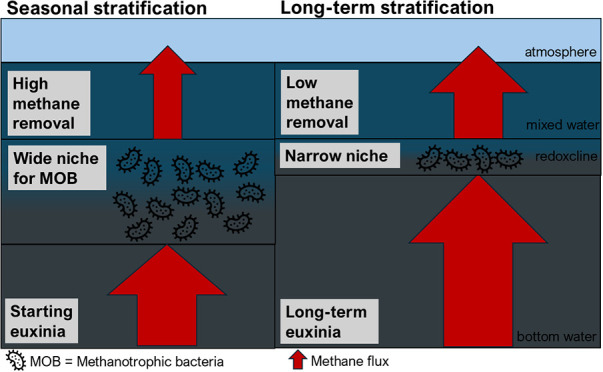

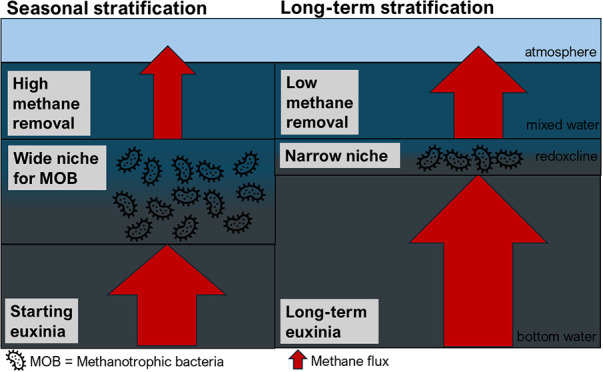

## Introduction

Coastal ecosystems are estimated to contribute up to 75% of global marine methane emissions, despite covering only 16% of the marine realm.^1,2^ Increasing exposure to eutrophication and deoxygenation,^3,4^ stimulates benthic methane fluxes, making microbial methane removal in the water column a crucial contributor to the mitigation of methane emissions.^5–7^ In stratified waters, aerobic methane-oxidizing bacteria (MOB) populate the oxygen-methane counter gradient.^8,9^ Genomic analysis of the MOB communities and laboratory experiments show versatile metabolic adaptations of MOB genera to a broad range of oxygen concentrations, which can extend the ecological niche of the MOB even into the anoxic water layers.^10–12^ There, the putatively aerobic methane oxidizers can potentially oxidize methane with alternative electron acceptors such as nitrate and metal oxides, contributing to water column methane removal even under anaerobic conditions.^10,12,13^ As coastal waters are particularly susceptible to prolonged stratification periods and resulting bottom water deoxygenation,^4,14^ such potential anaerobic methane removal may become a more important process in mitigating methane emissions in the future. However, longer-term stratified marine basins are not only exposed to deoxygenation, but also to the accumulation of sulfide (euxinia). However, it is still unclear how altered stratification regimes and associated euxinia impact water column methane removal in these systems.

In a stratified basin, the bottom layer is isolated from the top layer by a transition layer (e.g., thermocline, redoxcline), and the availability of reduced compounds is determined by the seasonality of organic matter supply into the bottom layer^15^ and the diffusive flux of reduced compounds from the sediment.^16,17^ Longer-term isolation of coastal bottom waters can ultimately lead to highly euxinic conditions and concurrently alter the entire microbial community composition resulting in simultaneous loss of microbial diversity and biogeochemical pathways.^18–20^ Additionally, in long-term stratified marine basins, the bottom waters can be populated by the benthic microbial community.^21^ Such a shift in microbial community composition and potential biogeochemical pathways during prolonged isolation can in turn affect the vertical distribution, activity and composition of the water column methanotrophic community. However, little is known about how this will affect the methane-filtering capacity of MOB in different layers of the coastal water column, and the persistence and composition of MOB communities in sites differentially affected by stratification.

Here, we investigated the methane biofilter in three basins of the inner Stockholm Archipelago with distinct stratification durations (seasonal and prolonged stratification) and associated different bottom water redox conditions (irregular, seasonal euxinia to longer-term euxinia). To elucidate the effect of longer-term stratification and anoxia on overall methane removal, and specifically the potential for methane removal from euxinic waters, we compared the methane flux, water column chemistry, MOB community composition, as well as network structure of the entire bacterial community in the water column of these three basins.

## Materials and Methods

### Fieldwork Location and Sampling Methodologies

The Stockholm Archipelago is a brackish ecosystem consisting of a network of basins with different shapes, sizes, and hydrological characteristics.^22^ The system has been reported to suffer from eutrophication due to high nutrient inputs from the catchment.^23^ Stratification and water column deoxygenation vary from basin to basin.^24^ Detailed descriptions of the sampling area are provided in ref 7,25 . Here, we selected three sites (Figure 1) with seasonal or longer-term stratification and corresponding bottom-water euxinia (Supporting Figure S7; Swedish Meteorological and Hydrological Institute: as reported in ref 7). In Skurusundet, the water column mixes approximately once every 2 to 4 years.^7^ This prolonged stratification leads to longer-term bottom water euxinia with high sulfide concentrations, as encountered during the time of sampling (bottom water O_2_/H_2_S < 2/349 μmol L^–1^ ^7^) (**SKS**; depth 26.5 m; N 59°17.902′; E 18°13.764′). In Stora Värtan (**VAR**; depth 29 m; N 59°24.252′; E 18°08.198′), the water column is seasonally stratified with recurring bottom water euxinia (bottom water O_2_/H_2_S < 2/43), while summer stratification in Södra Växholmsfjärden (**VAX**, depth 24.5 m; N 59°23.934′; E 18°20.796′) leads to seasonal bottom water deoxygenation, but with less severe and more irregular euxinia (bottom water O_2_/H_2_S 81/0 μmol L^–1^; see Supporting Figure S7 based on Swedsh Meteorological and Hydrological Institute: as reported in ref 7).

**Figure 1:**
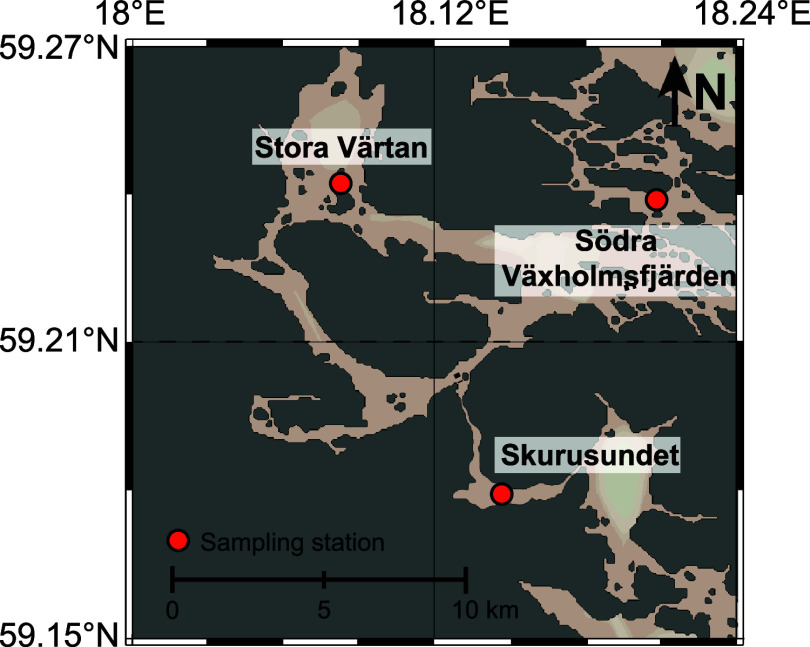
Map showing the three study sites Södra Växholmsfjärden (**VAX**; irregular euxinia), Stora Värtan (**VAR**; seasonal euxinia), and (Skurusundet **SKS**; longer-term euxinia) in the inner Stockholm Archipelago. Adapted with permission from ref 7. Copyright [2024] [Environ Sci Technol].

The water column at the three sites was sampled in a high vertical resolution during a cruise with the R/V Electra in September 2022. Stratification was identified based on the continuous measurement of oxygen, salinity, temperature, and depth in the water column with a CTD unit (SBE 911 plus, Sea-Bird Electronics Bellevue WA, USA). The top, bottom and transition water layers were determined based on the position of the thermocline and oxycline (Supporting data 1). Water below the thermocline was assumed to be the most isolated but still homogeneous. Water above the oxic threshold (>63 μmol L^–1^) in the oxycline in VAR and SKS was defined as the top water layer. The water in between these layers was defined as the transition zone. In VAX, oxygen concentrations did not go below 63 μmol L^–1^ (Figure 2C). Samples for determination of methane, methane oxidation rates and DNA extraction were taken as described in the Supporting Methods S1.4.

**Figure 2:**
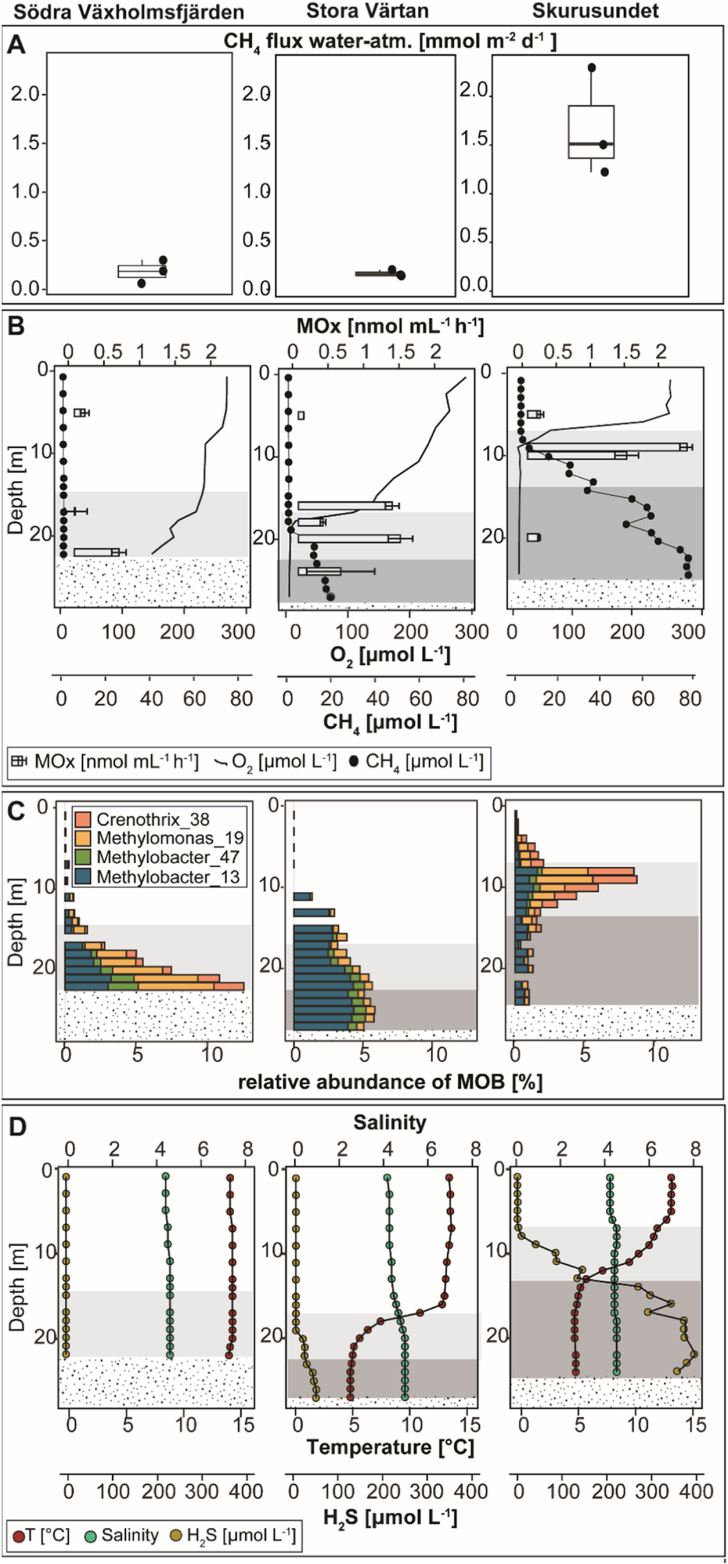
In situ water–air methane fluxes and water column depth profiles for the irregularly VAX (left), seasonally VAR (middle), and longer-term euxinic site SKS (right) in the Stockholm Archipelago in late summer 2022. (A) Measured in situ methane fluxes from the water column into the atmosphere. Boxes indicate the first and third quartiles, lines indicate the median, whiskers indicate outer data points if less than 1.5 interquartile range from quartiles, *n* = 3. (B) Depth profiles of temperature, salinity and sulfide, (C) oxygen, methane and potential methane oxidation rates and (D) relative abundance of 16S rRNA gene amplicon sequences belonging to MOB. In graphs (B–D), top, transition, and bottom water are indicated with unshaded, light gray and dark gray shading, respectively. The dotted area indicates sediment to illustrate the different depths of the sampling points.

### Methane and Sulfide Concentration Measurements

Methane concentrations in the headspace of the sampling bottles were measured with a Thermo Finnigan Trace gas chromatograph with a Flame Ionization Detector. To create a headspace, 10 mL samples were replaced by 10 mL N_2_ gas and equilibrated for a minimum of 2 h before measurement. Methane concentrations in the samples were then calculated by Henry’s law (Supporting Methods S1.1). Sulfide concentrations were determined using the phenylenediamine and ferric chloride method.^26^

### Potential Methane Oxidation Rates

To test potential aerobic methane oxidation rates, we incubated samples from each site and water layer. Specifically, incubations were set up for the mixed layer, the oxygenated top layer, the bottom layer below the thermocline and the transition zone between the top layer and the isolated bottom layer (Supporting Data 1). For each site and water layer, 100 mL of sample was transferred into a 120 mL borosilicate serum bottle, under an ambient air, closed with bromo-butyl stoppers and crimp-capped with an aluminum lid. Labeled methane (^13^C–CH_4_, 99%) was added to each incubation. The incubations were stored in the dark at room temperature under constant shaking (150 rpm). For each time point, 1 mL of liquid sample was removed from the incubation bottle and was subsequently replaced by 1 mL of air. To prevent under or overestimation of ^13^C–CO_2_ production, due to pH-sensitive changes in the carbonate balance, we acidified the subsamples with 50 μL of 0.1 mM HCl to a pH of less than 3 into a 3 mL, gastight vial (Labco, exetainer, UK). ^13^C–CO_2_ in the headspace of the subsamples was measured with a GC-MS (Agilent 5975C inert MSD). Using the Henry’s Law coefficient, the total amount of ^13^CO_2_ in each subsample was calculated (Supporting Methods S1.1). Potential methane oxidation rates were calculated as a linear regression of the ^13^C–CO_2_ increase over time.

### In Situ Methane Fluxes

Water–air methane fluxes were measured at each site, in the early afternoon (1–2 pm). Fluxes were measured with a LICOR trace gas analyzer (LI-7810, LI-COR Environmental—UK Ltd., Cambridge, UK) connected to a transparent, cylindrical floating chamber (ø: 390 cm, height 27 cm, TechnoCentrum, Radboud University, Nijmegen, NL) as described in ref 12.

Methane fluxes were calculated based on three replicate measurements per site using the following equation

$${\mathrm{CH}}_{4}\;{\mathrm{atmospheric}\;\mathrm{flux}}_{\mathrm{in}‐\mathrm{situ}}=\frac{{\Delta \mathrm{CH}}_{4}}{\Delta t}\times \frac{V}{A}$$

where ΔCH_4_/Δ*t* is the linear increase of the concentration of methane (mmol m^–3^) in the chamber over time (Δ*t*). *V* is the volume of the chamber (m^3^) and *A* is the area of the chamber (m^2^). The measured methane partial pressure (ppb) in the chamber was converted to methane concentrations (mmol m^–3^) using the ideal gas law and the ambient air temperature during each deployment.

### Microbiome Analysis and Statistical Methods

DNA was extracted following the FastDNA SPIN Kit for soil DNA isolation Kit (MP Biomedicals) and stored at −20 °C until further processing. The composition of the microbial community was assessed by 16S rRNA gene amplicon sequencing (Illumina MiSeq platform, Macrogen, Amsterdam, The Netherlands) with the primer pairs Bac341F (CCTACGGGNGGCWGCAG),^27^ Bac806R (GGACTACHVGGGTWTCTAAT);^28^ for bacteria, and Arch349F (GYGCASCAGKCGMGAAW) with Arch806R (GGACTACVSGGGTATCTAAT) for archaea.^29^ The data were analyzed with DADA2,^30^ taxonomy assignment of amplicon sequence variants (ASVs) was inferred from the Silva v138 database, and MEGA-11 ^31^ was used for phylogenetic tree construction as described in the Supporting (Section S1.2).

Diversity indices, the dissimilarity between samples and correlation with environmental factors (O_2_, CH_4_, H_2_S, SO_4_^2–^, NH_4_^+^, temperature and salinity) were calculated with the *vegan* package.^32^ Nonmetric multidimensional scaling (NMDS) was used to illustrate the community dissimilarity between samples and the correlation between change in environmental factors ([O_2_]_aq_ [CH_4_]_aq_, [H_2_S]_aq_, [SO_4_^2–^]_aq_, [NH_4_^+^]_aq_, temperature and salinity) and microbial composition was calculated with the *env*_*fit*() function (Supporting Methods S1.2).

To test which taxa significantly differed in relative abundance between the top, bottom and transition layer at each site, we conducted a differential abundance analysis of taxa with a relative abundance higher than 0.5% with a Wilcoxon test (BH) corrected; *p*-value <0.05, *n* = 5–10 (see Supporting Tables S2 and S3) by run_aldex­()^33^ modified based on ALDEx2.^34^ Moreover, all *p*-values are corrected for multiple testing using Benjamin–Hochberg procedure. To illustrate the co-occurrence of taxa integrated overall water layers, Levin’s niche overlap levins.overlap­() function; *MicroNiche* ^35^ was calculated between taxa with a higher abundance than 0.5% and prevalence in at least 10% of all samples at each site.

### Metagenomic Analysis

To investigate the niche specificity of the MOB community, we sent samples for full metagenomic sequencing (Illumina DNA PCR-Free (450 bp insert) low input kit, Novaseq platform, Macrogen, Amsterdam, The Netherlands). For the longer-term euxinic site SKS, we sent representative samples from the bottom (19 m), the top (6 m) and the transition zone (9 m). For VAR, we sent samples from the top (16 m) and transition zone (18 m) and for the mixed site VAX, we sent one representative sample from the peak with high MOB 16S rRNA amplicon abundance at 21 m. Metagenomic raw reads were assessed and processed by an adapted in-house pipeline “binmate” as described in in ’t Zandt et al., 2019, see Supporting Section S1.3. For MAGs taxonomically assigned as methanotrophic bacteria (MOB MAGs) a phylogenomic tree, based on gtdb-tk reference genomes, was generated using the interactive Tree of Life, iTOL v6 (https://itol.embl.de) (Supporting Section S1.3 and Figure S10). Finally, annotation of the bins, the unbinned fraction, and sequences shorter than 2500 bp was performed using *Metascan*.^37^ An overview of genes of relevant pathways in the different water layers and sites was generated based on gene read counts according to *Metascan* output. Gene read counts were transformed into reads per kB million (RPKM) and the coverage was calculated using *coverM* v0.7.0. The coverage data were then merged with the annotated genes with an *E*-value lower than 0.001. Gene abundance was visualized in R with the *ggplot2*. The raw metagenomic reads are accessible on NCBI PRJNA1126564.

### Co-Occurrence Network Analysis

To investigate the correlations between MOB and other microbial community members and elucidate differences in the top, bottom and transition water layers, we conducted network co-occurrence analysis with the *NetCoMi* package.^38^ To emphasize the representation of the diversity of abundant taxa, Pearson correlations were calculated only with taxa with a higher total abundance of 0.5% of all samples (5–10, Supporting Table S2) per layer. *P* values were adjusted by local false recovery rate correction implemented in the *netConstruct­()* function of *NetCoMi*.^38–40^ A high distance for strongly negative correlations between taxa was assured by applying the “signed” distance metric for the transformation into dissimilarities. For better visibility of relevant connections (sparsification), we used a correlation threshold of >0.3 before transformation into the adjacent matrix. To avoid a compositional effect by the Pearson correlation, read counts were transferred into Euclidean space by center log-ratio transformation (clr).^41^ Clusters were identified by the cluster_optimal algorithm, and centralities were calculated by eigenvector decomposition. Betweenness centrality was used to define hubs, in which nodes are defined as a centrality value above the empirical 95% quantile. To quantify the cluster connectivity, we calculated the ratio of the number of between-cluster edges to the number of edges within each cluster. For network visualization, colors indicate clusters and eigenvectors correspond to the node size. Hubs are indicated with black node borders and bold text.

## Results

### Highest Methane Accumulation and Water–Air Fluxes in Longer-Term Euxinic Site

The oxygen and methane availability at the three sampling stations was compared by creating high-resolution profiles (O_2_, CH_4_, H_2_S, temperature, salinity) of the water column in September 2022 (Figure 2B,C). In VAR and SKS, the CTD and oxygen profiles show thermal stratification and anoxic bottom waters. However, the depth and extent of the thermocline and oxycline differed. At SKS, hypoxic conditions were already reached at 7 m depth (40 μmol L^–1^, top water boundary), and oxygen was not detected from 8.4 m downward until the sediment at 22 m. The lower boundary of the thermocline at 14 m was used as the start of the bottom water layer. Sulfide was present throughout the oxygen-depleted waters reaching concentrations of 370 μmol L^–1^ close to the sediment-water interface. The bottom water–methane concentrations were very high reaching 84 μmol L^–1^, and the methane concentrations linearly decreased until the oxycline and then decreased exponentially. However, the methane concentrations in the top layers remained high (0.3–0.4 μmol L^–1^), which was reflected in the highest measured diffusive water–air methane fluxes at this site (1.68 mmol m^–2^ d^–1^ (±0.02)) (Figure 2B,A, respectively). At VAR, the oxycline vertically expanded between 11–19 m and hypoxic conditions (<63 μmol L^–1^) at 17 m determined the lower boundary for the top water layer. Water below the thermocline (>22 m) was defined as the bottom water layer. Although sulfide accumulated in the bottom water, the maximum concentrations (44 μmol L^–1^) were almost 10 times lower than at SKS. The exponential decrease of methane concentrations at VAR started below the oxycline (20 m) and mean methane fluxes were 0.13 mmol m^–2^ d^–1^ (±0.09) (Figure 2A). Oxygen concentrations remained 128 μmol L^–1^ at the sediment-water interface in VAX and no sulfide was detected. Water above the oxycline at 15 m was determined as the top layer, and below as the transition zone. There, methane concentrations were much lower than in SKS and VAR and fluctuated between 0.3 to 0.5 μmol L^–1^ and decreased gradually from 7 m to the surface water (0.1 μmol L^–1^). Yet, water–air fluxes were slightly higher than in VAR (0.19 mmol m^–2^ d^–1^ (±0.01) (Figure 2A)).

### Vertical Distribution of Potential Aerobic Methane Removal

We observed active aerobic methane removal in all incubated samples from all sites and water layers. In VAX methane oxidation rates were much higher in the transition zone near the sediment-water interface, than in the top layer (0.63, 0.01, and 0.15 μmol L^–1^ h^–1^ respectively, Figure 2C). In the seasonally euxinic site VAR, methane oxidation rates were lowest in the fully oxic top water at 5 m depth. In the bottom water, the highest methane oxidation rates were measured at 16 and 20 m depth (1.33 and 1.44 μmol L^–1^ h^–1^ respectively). At the longer-term stratified and euxinic SKS, methane oxidation rates were highest at 9 m (2.35 μmol L^–1^ h^–1^) and lower with 1.47 μmol L^–1^ h^–1^ at 10 m where oxygen was depleted and methane accumulated.

### Vertical Shift of MOB Community Composition along Redox Gradients

The methanotrophic community at all three sites was dominated by MOB belonging to γ-proteobacteria (γ-MOB). Other methanotrophic microorganisms such as methanotrophic archaea, verrucomicrobial NC10 or α-proteobacterial MOB were not found in significant abundance (<0.1% of amplicons) in the water column. Phylogenetic analysis showed that the ASVs assigned to *Methylomonas* and *Methylobacter* grouped with several different species (Supporting Section S2.1 and Figure S6). The vertical distribution pattern of MOB relative abundances in the water column differed between the sites (Figure 2D). In the fully oxic site VAX, the MOB community peaked at the bottom of the basin, with a relative abundance of 13%, and decreased exponentially toward the surface with decreasing relative abundances of *Methylobacter_ASV_47* and *Crenothrix_ASV_38*, but *Methylobacter_ASV_13* and *Methylomonas_ASV_19* increased in relative abundance. At the stratified site VAR, the relative abundance of MOB was very low in the top layer, increased from 0.01% at 7 m to 5% at 21 m along the oxycline, and remained between 4 and 6% in the bottom with *Methylobacter_ASV_13* dominating. *Methylomonas_ASV_19* and *Methylobacter_ASV_47* remained below <1% throughout the water column, and C*renothrix_ASV_38* was not detected in VAR. In the highly sulfidic, long-term stratified site SKS, the MOB relative abundance peaked in the transition zone (8% rel. abundance) and decreased toward the top and the bottom water layer. *Methylomonas_ASV_19 and Crenothrix_ASV_38* were dominant in the top water. Both *Methylobacter* ASVs were less abundant throughout the oxycline. However, *Methylobacter_ASV_13* dominated in the bottom water (Figure 2D).

### Metagenome-Assembled Genomes of Methanotrophic Bacteria

To investigate the genomic versatility of the MOB community in the different water layers, we sequenced DNA from the top, bottom and transition water layers in SKS, from the top and the transition layer in VAR and the depth with the peak in MOB relative abundance in VAX. From 168 bins, we retrieved four metagenome-assembled genomes (MAGs) that were taxonomically classified as distinct genera belonging to *Methylomonadaceae* and were >63% complete and <4% contaminated and mapped to reads per MAG (Tables 1 and 2). Based on the genome taxonomy database *gtdb-tk* MAG_18 was identified as *Methylobacter*, MAG_130 as *Methylomonas*, MAG_184 as *Methylovulum* and MAG_264 as *KS41* (*Methylobacter*) (hereafter referred to as MAG_18_*Methylobacter*, MAG_130_*Methylomonas*, MAG_184_*Methylovulum* and MAG_254_*KS41* respectively).

**Table 1:** Completeness, Contamination, and Redundancy of the Retrieved MOB MAGs

MAG_name	completeness [%]	contamination [%]	redundancy [%]
MAG_18_Methylobacter	70.0	3.3	66.7
MAG_130_Methylomonas	63.4	3.5	50.0
MAG_184_Methylovulum	83.5	0.5	40.0
MAG_254_KS41	68.9	3.6	70.6

**Table 2:** Mapped Reads per MOB MAG [%], of Each Sample

site	depth [m]	water layer	MAG_18_Methylobacter	MAG_130_Methylomonas	MAG_184_Methylovulum	MAG_254_KS41
SKS	6	top	0.05	0.46	0.47	0.12
SKS	9	transition	0.15	1.84	1.68	0.54
SKS	19	bottom	0.04	0.29	0.01	0.33
VAR	16	transition	0.14	0.30	0.00	0.73
VAR	19	bottom	0.31	0.48	0.00	0.95
VAX	21	transition	0.90	2.04	0.93	1.07

While MAG_254_*KS41* and MAG_184_*Methylovulum* contained *pmoABC*, MAG_130_*Methylomonas* and MAG_18_*Methylobacter* did not contain any *pmo*. However, *pmoABC* gene sequences were found in the unbinned fraction, of which *blastp* revealed that *pmo* gene sequences JNEEOHEB_334838–334840 and JNEEOHEB_289957–289959 had a high identity match (>98%) to *Methylomonas lenta* sequences, and *Methylobacter sp*. *g*ene sequences (Supporting Data 2). Next to the particulate methane mono-oxygenase genes, MAG_184_*Methylovulum* also contained the gene cluster of a soluble methane mono-oxygenase. MAG_130_*Methylomonas*, MAG_184_*Methylovulum*, and MAG_254_*KS41* all contained the gene for the lanthanide-dependent methanol dehydrogenase (*xoxF*) and MAG_18_*Methylobacter* contained the calcium-dependent methanol dehydrogenase (*mxa*). However, we also found an *mxa* gene in the unbinned fraction of which *blastp* revealed a high similarity to *M. lenta* (Supporting Data 2). Several marker genes for genomic adaptations to low oxygen concentrations were present in all MOB MAGs (Figure 3). For instance, genes for (partial) denitrification were found in MAG_130_*Methylomonas*, which had a high percentage of mapped reads in the transition and bottom water. High-affinity cytochrome bd ubiquinol oxidase marker genes (*cydAB*) were present in all four MOB MAGs. Except for MAG_254_*KS41*, various marker genes for fermentation processes and nitrogen fixation were also present in all MOB MAGs (Figure 3).

**Figure 3:**
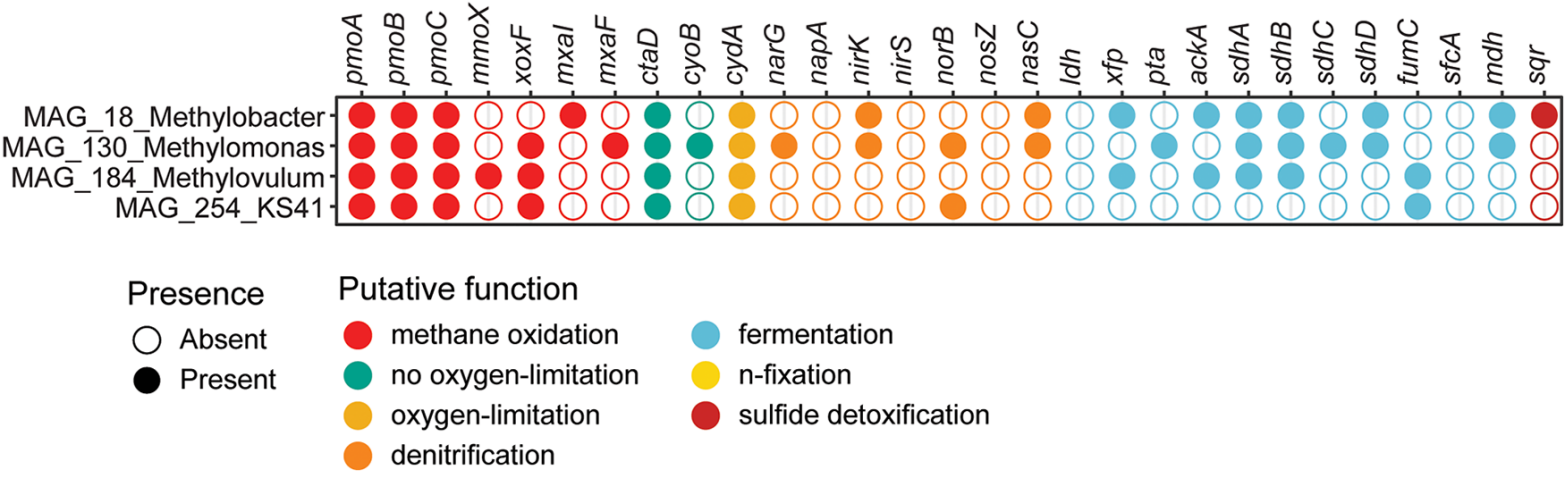
Presence and absence of genes involved in methane oxidation (particulate methane monooxygenase) (*pmoABC*), soluble methane monooxygenases (*mmoX*), lanthanide dependent (*xoxF*) and calcium dependent (*mxaI,F*) methanol-dehydrogenase, low-affinity oxidases (*catD*, *cyoB*), high-affinity oxidases (*cydA*), as well the denitrification genes membrane-bound nitrate reductase (*narG*), periplasmic nitrate reductase (*napA*), nitrite reductase (*nirK/S*), nitric oxide reductase (*norB*), nitrous oxide reductase (*nosZ*), and nitrogen fixation (*nifD/nifK*). Finally, we included key genes for enzymes involved in fermentation such as lactate dehydrogenase (*ldh*), phosphate acetyltransferase (*pta*), acetate kinase (*ackA*), succinate dehydrogenase (*sdhABCD*), fumarate hydratase (*fumC*), NAD-dependent malic enzyme (*sfcA*), malate dehydrogenase (*mdh*), aconitate hydratase (*acnAB*), and sulfur oxygenase/reductase (*sqr*).

### Microbial Diversity and Microbial Network Structure in Different Water Layers

The microbial community compositions in top water samples of all three sites were similar and formed a broad cluster in the NMDS analysis (Supporting Figure S3). In contrast, the community dispersal between samples of the transition layer was high and resulted in very distinct communities in the bottom water layers of SKS and VAR. This dispersal was correlated with oxygen, temperature and salinity at VAR and with sulfide and methane in SKS (Supporting Table S4). In general, the relative abundances of Cyanobacteria, Actinobacterota, Bacteroidota, Verrucomicrobiota and Planctomycetota decreased and Campylobacterota and Pseudomonadota increased moving from top to bottom layers. Notably, in SKS Desulfobacterota increased the most between the transition and bottom layer (Supporting Table S3).

The Pearson correlation-based co-occurrence networks of samples grouped by site and water layer, illustrated the differences in community connectivity and importance of certain taxa (Figure 4 and Supporting Figure S8, Supporting Section 2.1). Most notably, in the stratified sites VAR and SKS, the ratio of positive edges between to within clusters was higher in the top layer compared to the bottom water layers (Supporting Table S5), and is also noticeable as general decrease in network complexity (Figure 4). In the bottom and transition water layers of VAR, MOB ASVs were predominantly connected to putative sulfur-oxidizing bacteria (belonging to Campylobacterota). However, in the bottom water of SKS, *Methylobacter_ASV_13* clustered with sulfate-reducing bacteria (Desulfobacterota). Moreover, this cluster was completely separated from the Campylobacterota cluster.

**Figure 4:**
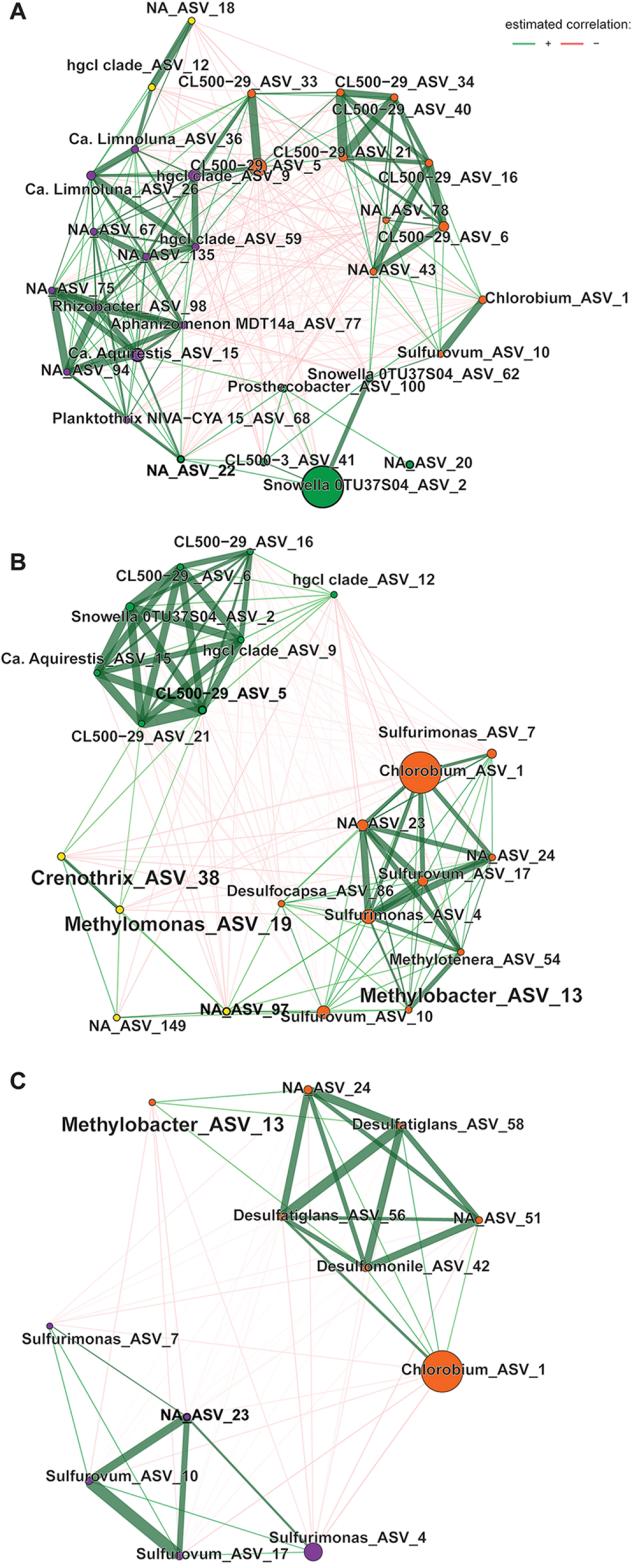
Co-occurrence networks based on Pearson correlation for (A) top, (B) transition and (C) bottom layers of SKS. Edges are displayed as green lines for positive correlations and red lines for negative correlations. Strong positive correlations are emphasized by line width. Node colors indicate clusters and node size indicates abundance. Hubs based on betweenness are indicated with black node borders and bold font. Methane oxidizing bacteria are highlighted by a bigger font.

Levin’s niche overlap illustrated a high co-occurrence of MOB with ASVs from the sulfur-cycling phylum Campylobacterota and with members of Pseudomonadota and low niche overlap with Cyanobacteria, Desulfobacterota and Verrucomicrobiota (Supporting Figure S4).

## Discussion

Microbial methane removal in the water column is a major control of methane emissions from coastal ecosystems.^42^ Although recent studies have shown high potential for bacterial methane removal even in anoxic and euxinic bottom waters,^12,43^ to what extent prolonged stratification and resulting shifts in bottom water redox conditions affect MOB-mediated methane removal potential is poorly understood. Here we compared the functioning of the methane biofilter in an irregularly, seasonally and long-term euxinic basin of the inner Stockholm. By analyzing microbial community composition, methane removal potential and water–air methane fluxes, we revealed strikingly higher water–air methane fluxes and lower potential methane removal, with longer-term euxinia.

### High Methane Accumulation and Water–Air Fluxes Sustained by a Lack of Microbial Methane Removal

Longer-term euxinia in SKS was accompanied by a high methane accumulation in the bottom water and the highest water–air methane fluxes. Measured diffusive water–air methane fluxes at all three study sites of the inner Stockholm Archipelago were high (0.13–1.68 mmol m^–2^ d^–1^) compared to other coastal ecosystems.^44,45^ The inner Stockholm Archipelago suffers from eutrophication and anoxia which leads to high diffusive methane fluxes from the sediment into the water column^46,47^ and increased methane ebullition.^7,48^ The variation of benthic methane fluxes at the longer-term euxinic, seasonally euxinic and fully oxygenated sites (2.2, 5.6, and 2.4 mmol m^–2^ d^–1^ respectively), was driven by the depth of the sulfate-methane transition zone (SMTZ) in the sediment.^7^ Although the benthic methane fluxes at the seasonally euxinic site (VAR) were more than twice as high, methane concentrations were still highest in SKS’s longer-term euxinic bottom water. There, the bottom water isolation allowed for the accumulation of methane and sulfide over a longer period (see Supporting Figure S7), which is often observed in long-term stratified waters.^16,17^ In addition, the potential methane oxidation rates were lower, and the vertical MOB distribution was narrower in the longer-term euxinic bottom water in SKS, compared to the seasonally euxinic bottom water in VAR (Figure 2C and Supporting Data 1). This lack of microbial methane removal in the bottom water further amplified methane accumulation. Prolonged stratification and euxinia could thus lower the microbial methane filter capacity through changes in MOB activity, diversity, and niche width and exacerbate methane emissions from stratified brackish waters.

### Drivers for Vertical Niche Distribution of MOB

MOB abundance is often high along the methane-oxygen counter gradient where both methane and oxygen supply are optimal.^49,50^ Accordingly, the relative abundance of MOB was high in the transition zone between the isolated bottom layer and the mixed top layer at all sites (Figure 2D). The MOB relative abundance declined much more toward the top than toward the bottom layers. However, vertical distribution and activity of microbes in the water column are determined by a combination of substrate availability and microbial interactions.^51–53^

Low MOB abundance in surface waters is an often observed phenomenon. Still, potential mechanisms limiting the MOB niche in highly turbulent surface waters, (e.g., low methane concentrations, light inhibition and feeding pressure) are controversially discussed.^54^ Although oxygen was abundant and methane concentrations were high enough for methanotrophy,^55,56^ a nonlinear correlation between oxygen concentrations and methane oxidation rates has been described previously.^57^ Moreover, competition for macronutrients with dominant heterotrophic Actinobacterota could be another important factor limiting MOB abundance.^5,58^ In the transition layer, slower diffusive processes limit oxygen and nutrient supply into the bottom water, which results in different biogeochemical niches for microbes and a vertical shift in the microbial community composition.^59–61^ Indeed, NMDS analysis (Supporting Figure S3) illustrated a high variation of the microbial community between transition layer samples, with a prominent shift away from photo- and heterotrophic bacteria toward Campylobacterota and Pseudomonadota, including MOB (Supporting Figure S1 and Table S3) and was concurrent with high RPKM values of genes involved in autotrophic carbon-fixation (rather than heterotrophy) in the samples of the bottom and transition water layers (Supporting Figure S5). Levin’s niche overlap of MOB ASVs was generally high with Campylobacterota and Pseudomonadota, but not with Cyanobacteria and not with the dominant heterotrophic Actinobacterota in the top layers (Figure S4). Experiments have shown that MOB are outcompeted by heterotrophic bacteria even if oxygen concentrations are high.^62^ Ubiquitous members of the Actinobacterota, such as the here abundant *CL500–29 marine group* and *hgcI clade*, can use a wide spectrum of organic compounds^63,64^ and could thus outcompete MOB in the top layer. In the transition and bottom layers, carbon incorporation via methane-oxidation intermediates might give an advantage in the CO_2_-fixation-dominated, shaded water layers (Supporting Figure S5). Moreover, MOB may better recover from oxygen-limiting conditions than from methane-limiting conditions. Methane limitation under aerobic conditions can force MOB to oxidize endogenous cell carbon which severely impairs their recovery rate and competitiveness.^65^ In contrast, during oxygen limitation, MOB can potentially use alternative electron acceptors such as nitrate or metal-oxides, or enter a dormant or fermentative state,^13,66,67^ which is less prone to biomass loss.^43,65^ Indeed, all MOB MAGs, except *KS41* contained genes involved in either denitrification, fermentation or oxygen-scavenging/high-affinity oxidases (Figure 3). This could support the presence of MOB in the euxinic bottom water compared to much lower relative abundances in the top layers.

Versatile metabolic adaptations to oxygen limitation^68–70^ support recent evidence for MOB activity under oxygen-limited conditions, resulting in niche partitioning and niche width expansion, ultimately enhancing the system’s methane filtering capacity.^5,50,71^ The fraction of *Methylomonas_ASV_19* in the MOB community varied little throughout the depths at all sites, while the fraction of *Crenothrix_ASV_38*, *Methylobacter_ASV_47* and *Methylobacter_ASV_13* shifted with depth (Figure 2D). Generally, *Crenothrix_ASV_38* and *Methylobacter_ASV_47* were more abundant at higher oxygen concentrations, while *Methylobacter_ASV_13* extended the MOB niche into both the bottom and top water layers, especially in VAR. *Methylobacter* species are dominant methane oxidizers in many anoxic waters and can potentially use alternative electron acceptors such as nitrate under oxygen-limiting conditions.^69,72,73^ Notably, the niches of the two *Methylobacter* ASVs did not completely overlap (Supporting Figure S4), indicating that the niche specificity of the *Methylobacter* ASVs likely differs within genera or at even finer taxonomic scales. Moreover, members of *Methylomonas* and *Crenothrix* also show adaptations to oxygen limitation, e.g., by their potential for nitrate reduction,^68,69,74^ but were not found in the euxinic bottom water in the inner Stockholm Archipelago. Thus, niche adaptations may be species-specific and can vary between environments, potentially depending on interactions within the microbial community. Still, the high relative abundance of *Methylobacter_ASV_13* in the euxinic bottom water of VAR but not in the longer-term euxinic SKS indicates potential limitations to MOB-mediated methane removal ultimately resulting in higher oceanic methane emissions with longer-term euxinic conditions.

### Long-Term Euxinia Changes Microbial Community Composition and Drives MOB Out of Bottom Water

In our study, the overall topology of correlation-based co-occurrence networks differed between the samples grouped by sites and water layers. Most notably, the network complexity of the bottom waters was lower than in the top layers, with fewer nodes, and higher isolation of the separate clusters (Figure 3 and Supporting Figure S8). This was most pronounced in the bottom layer of SKS which was isolated from the top layer for a prolonged period.^7^ In such longer-term euxinic waters, most electron acceptors are depleted and the microbial community is typically dominated by sulfur-cycling Desulfobacterota.^19,60,75^ In VAR and VAX, the main sulfur-cycling microorganisms belonged to Campylobacterota and Pseudomonadota while in SKS, sulfide-oxidizing Chlorobia and sulfate-reducing Desulfobacterota were dominant. Sulfur-oxidizers of the phylum Campylobacterota (e.g., *Sulfurimonas*, *Sulfurovum*) are microaerophilic microorganisms that can oxidize reduced sulfur compounds to sulfate with oxygen or nitrate as an electron acceptor.^76^ Chlorobia are anaerobic, phototrophic sulfur-oxidizing bacteria often found in anoxic waters with low light availability.^77,78^ The community assembly in the longer-term euxinic bottom water could be driven by succession toward a fermenting community under persisting euxinia, which might support a shift from lithotrophic sulfur-oxidizers (e.g., Campylobacterota) to heterotrophic sulfate-reducing bacteria (e.g., Desulfobacterota) because Desulfobacterota cannot assimilate complex carbon and may require a fermenting community which can supply labile carbon.^20^ The succession toward a Chlorobia and Desulfobacterota-dominated community could hence be attributed to prolonged isolation and availability of easily biodegradable compounds and sulfate.^78^ Moreover, Desulfobacterota are sulfur-reducing bacteria that are often found in the sediment, together with methanotrophic archaea. If the water column is stratified over a longer period, the bottom water could become colonized by members of the sediment community (“seed bank” communities).^20,21^ Indeed, the relative abundance of methanogenic archaea that usually inhabit the anoxic sediment was high in the bottom water of SKS (Supporting Figure S2). This could point to such sediment-derived colonization of the bottom water. Yet, we did not find any methanotrophic archaea (ANME) in the amplicon sequencing analysis or *mcr* genes in the metagenome in the water column (Supporting Figure S2), and thus they likely did not contribute to anaerobic methane removal. The absence of ANME in the bottom water could be explained by their absence in highly sulfidic top sediments,^79^ which makes the colonization of the bottom water less likely. Together this implies that the ongoing succession and potential colonization by the sediment community in the isolated bottom water resulted in a distinct bottom water community at the two stratified sites.

A shift in the sulfur-cycling community induced by longer-term euxinia, could have limited the extension of MOB into the bottom layer and therefore overall vertical distribution and net methane-oxidation potential. The relative abundance of MOB in the bottom water was much lower in the sulfate-reducer-dominated bottom water of SKS than in the sulfur-oxidizer-dominated bottom water of VAR. Consistently in VAR, the low niche overlap of MOB with members of Desulfobacterota also indicates that MOB and Desulfobacterota are mutually exclusive. Such a recession of MOB co-occurring with a shift from sulfur-oxidizers to sulfate reducers in the bottom water has been observed previously.^20^ This further supports that deterministic processes, such as the simultaneous shift in redox conditions and microbial community induced by longer-term euxinia were the main factors leading to different methanotrophic community assembly and distribution in the two stratified sites. The co-occurrence networks showed a strong positive correlation between MOB (especially *Methylobacter_ASV_13*) and Campylobacterota in the redoxcline of all sites. Campylobacterota could have a positive effect on MOB under oxygen limitation, because Campylobacterota’s sulfide oxidizing activity may prevent sulfide accumulation and hence sulfide toxicity.^79,80^ Moreover, it may supply oxidized sulfur compounds that can be used by MOB during oxygen limitation (e.g., thiosulfate oxidation).^70^ Although MOB can potentially use a variety of alternative electron acceptors under anoxic conditions, longer-term stratification, especially in eutrophic coastal waters, can lead to a loss of electron acceptors which results in a shift toward fermentative pathways as discussed above. The combination of a loss of alternative electron acceptors and the population of the bottom water specialized in the use of locally available substrates such as sulfide, could increase the competition pressure on MOB, and drive MOB out of the bottom water limiting their vertical distribution and thus substantially lowering MOB-mediated methane removal.

## Environmental Implications

Even though the MOB community was active and vertically broadly distributed in the seasonally euxinic conditions, methane-removal capacity in longer-term euxinic conditions was considerably lower in our study. We suggest that a shift toward longer-term euxinic conditions can simultaneously alter the bottom water microbial community composition, which may force MOB away from the bottom water. This inherently reduces the spatial breadth of the microbial methane biofilter, drastically limiting its overall methane removal capacity. Hence, though methanotrophs have been shown to have versatile capacities to adapt to deoxygenation,^13^ prolonged stratification can lead to a loss of methane removal capacity in the water column.

Nutrient trapping, sea level rise and global warming have been previously identified as key drivers for the expansion of euxinia in the ocean’s history.^81^ With ongoing eutrophication,^82^ deoxygenation^83^ and global mean sea level rise in the 21st century,^84^ periods of coastal euxinia are likely expanding in the future.^4,14^ Our study emphazises the importance of understanding how longer-term euxinia affects methane mitigation, as the spatial and temporal heterogeneity of deoxygenation and euxinia is mostly overlooked.^85^ Our data shows that the duration of the stratification period and intensity of water column euxinia are crucial factors to consider in oceanic methane emission estimates and for assessing the impact of global change on coastal methane emissions.

While studies have shown that the water column MOB community is resilient to seasonal changes in water column chemistry,^12,86^ our study identifies longer-term euxinia as a threat to the functionality of the microbial methane filter; longer-term euxinia can induce loss of microbial diversity and metabolic pathways, potentially pushing the MOB community toward a tipping-point where functionality may be irreversibly lost.^87^ Some coastal waters might therefore be at high risk of reaching hard limits—when no adaptive actions are possible to avoid intolerable risks - even below 1.5 °C of global warming.^84^ Together with increasing benthic methane release with eutrophication,^7^ methane emissions from eutrophic coastal ecosystem will further increase in the future. It is therefore paramount for policymaking to further reduce the eutrophication of coastal ecosystems.

## Supplementary Material











## Data Availability

Raw reads of the metagenome sequencing data can be accessed on the NCBI under the accession number PRJNA1126564.

## Notes

Detailed description of of CO_2_ concentration calculations; 16S rRNA amplicon and metagenome sequencing analysis and methane flux measuremens. Supporting results of relative abundances of bacterial families and archaeal genera retrieved from 16S amplicon sequencing, α and β diversity indeces; niche overlap of bacterial families; overview of carbon fixation and sulfur cycling genes in the metagenome of samples of different water depths; long-term monitoring data of bottom water oxygen and sulfide concentrations of all three basins; co-occurrence networks and phylogeny of MOB (PDF)
MOB relative abundances (XLSX)
MOB unbinned blast and unbinned sequences (XLSX)
Sulfur cygling genes (XLSX)
Tree mapping and conserved genes (XLSX)
