# Supplementary material for: Long-Term Euxinia Restricts Microbial Methane Removal in Eutrophic Coastal Basins
Source: Environ Sci Technol. 2025 Oct 8;59(41):21988–2000. doi: 10.1021/acs.est.5c05066 (PMC12550815; doi:10.1021/acs.est.5c05066)
Supplement: Supplementary file 1 [file es5c05066_si_001.pdf]

Supporting Information

Long-term euxinia restricts microbial methane removal in eutrophic coastal basins

Short title: **Prolonged euxinia affects methane biofilter**

Keywords: tipping-point, long-term stratification, methanotrophic bacteria, anoxia, microbial community

Authors, email and affiliations:

Venetz, Jessica*[1], jessica.venetz@outlook.com, https://orcid.org/0000-0002-9654-0532

Dotsios, Nicky[1], nicky.dotsios@ru.nl, https://orcid.org/0009-0000-3938-197X

Żygadłowska, Olga M. [1,2], olga.zygadlowska@ru.nl, https://orcid.org/0000-0003-1457-4454

Lenstra, Wytze K. [1,2] wytze.lenstra@ru.nl, https://orcid.org/0000-0003-0979-5594

van Helmond, Niels A.G.M. [1,2], niels.vanhelmond@ru.nl, https://orcid.org/0000-0003-0024-7217

Humborg, Christoph [3], christoph.humborg@su.se, https://orcid.org/0000-0002-0649-5599

McMahon, Katherine D.[4], trina.mcmahon@wisc.edu, https://orcid.org/0000-0002-7038-026X

in ’t Zandt, Dina [5,6], dina.intzandt@ru.nl, https://orcid.org/0000-0001-6487-0273

Slomp, Caroline P. [1,2], caroline.slomp@ru.nl, https://orcid.org/0000-0002-7272-0109

Jetten, Mike S.M. [1,] mike.jetten@ru.nl, https://orcid.org/0000-0002-4691-7039

.Veraart, Annelies J [5], annelies.veraart@ru.nl, https://orcid.org/0000-0001-6286-7484

[1]*Department of Microbiology, Radboud Institute for Biological and Environmental Sciences, Radboud University, 6500 HC Nijmegen, The Netherlands*

[2]*Department of Earth Sciences, Faculty of Geosciences, Utrecht University, 3584CB Utrecht, The Netherlands*

[3]*Baltic Sea Centre, Stockholm University, SE 106 91 Stockholm, Sweden*

[4]*Departments of Civil and Environmental Engineering, and Bacteriology, University of Wisconsin- Madison, US 53706-1314 Madison, WI, USA*

[5]*Department of Ecology, Radboud Institute for Biological and Environmental Sciences, Radboud University, 6500 HC Nijmegen, The Netherlands*

[6]*Terrestrial Ecology, Netherlands Institute of Ecology, 6700 HB Wageningen, The Netherlands*

Corresponding author:

Dr. Jessica Venetz

Radboud Institute for Biological and Environmental Sciences (RIBES)

Radboud University, Nijmegen (NL)

Email: jessica.venetz@outlook.com

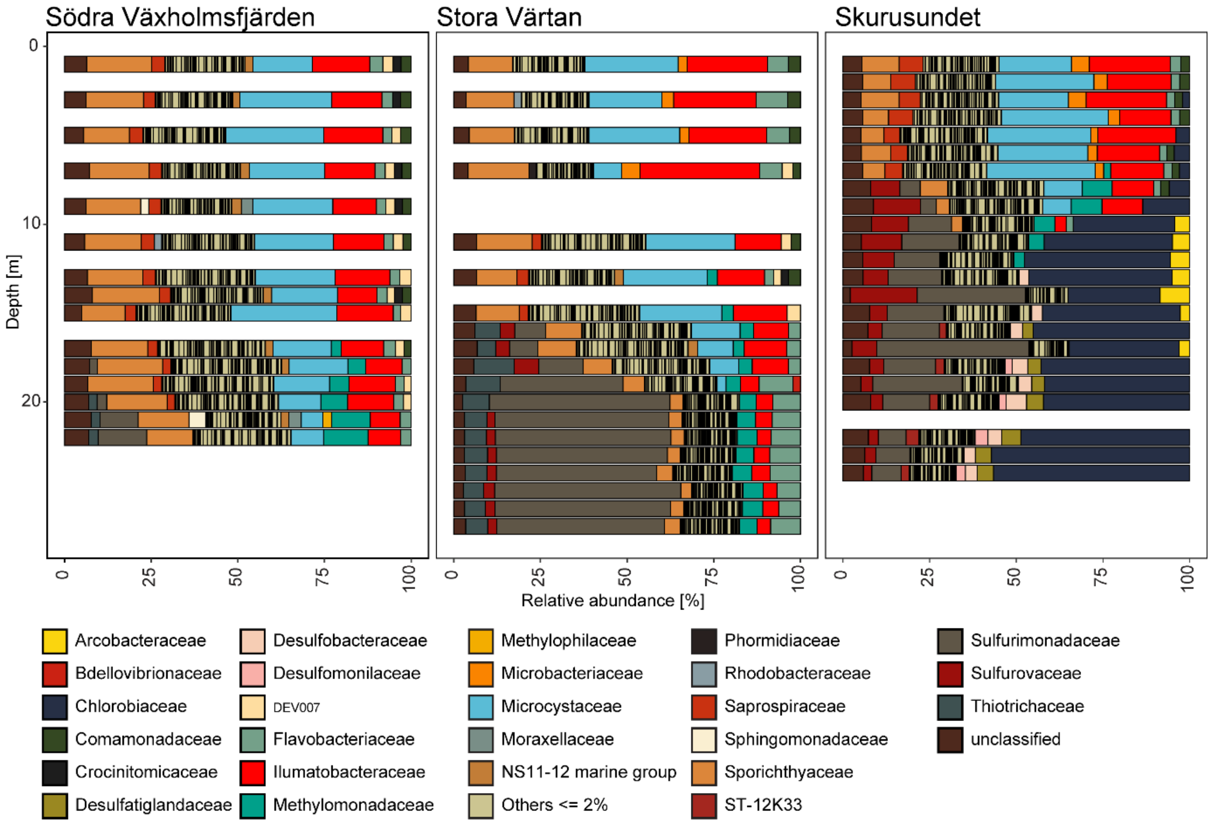

Figure S1: *Relative abundance of families with a relative abundance of > 2 % of all bacteria retrieved through 16S rRNA sequencing.*

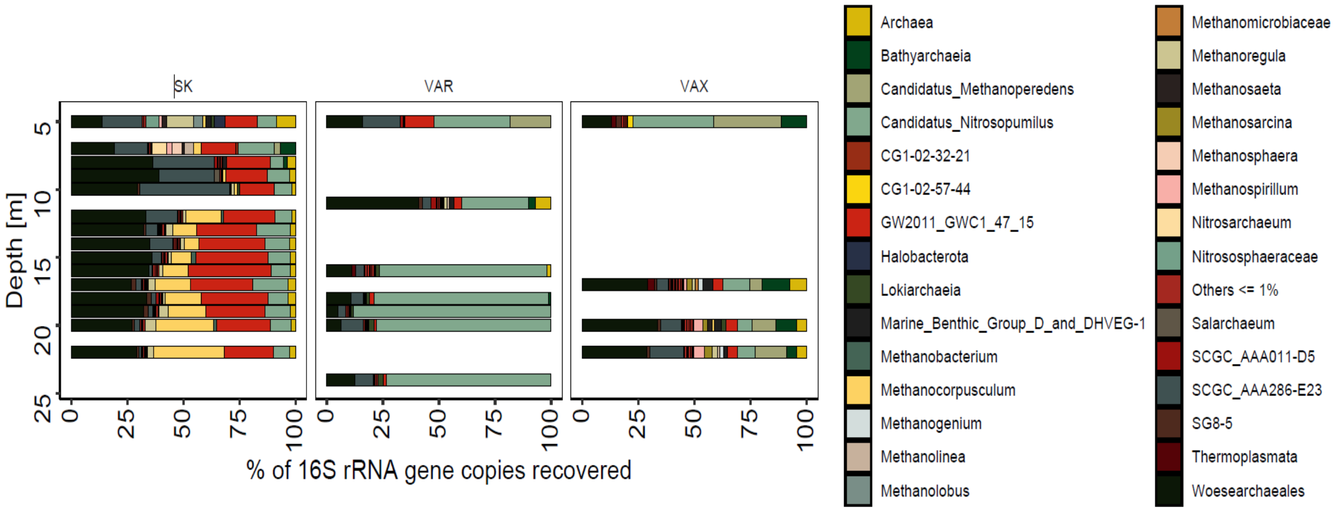

Figure S2: *Relative abundance of archaea retrieved through 16S rRNA sequencing (>1 %).*

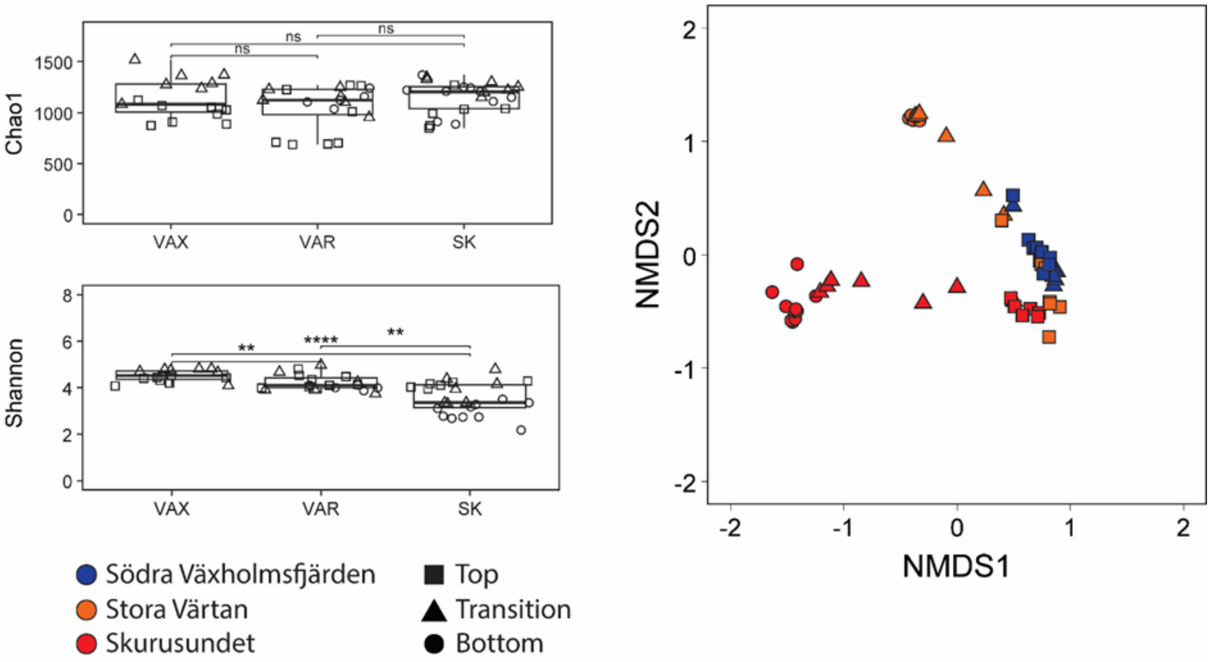

Figure S3: *Alpha diversity measures Chao1 (richness) and Shannon (richness and evenness) of the total water column bacterial community of the sampling site (left). Circles indicate bottom layer, triangles transition zone and squares top layer*. Asterix indicate p values for significance (**** $p < 0.0001$, ** $p < 0.01$, ns = not significant, $p > 0.05$). *Beta diversity of all samples calculated as Bray-Curtis distance and ordinated via two-dimension NMDS* (right) ($n_{VAX} = 15$, $n_{VAR} = 19$ and $n_{SKS} = 23$).

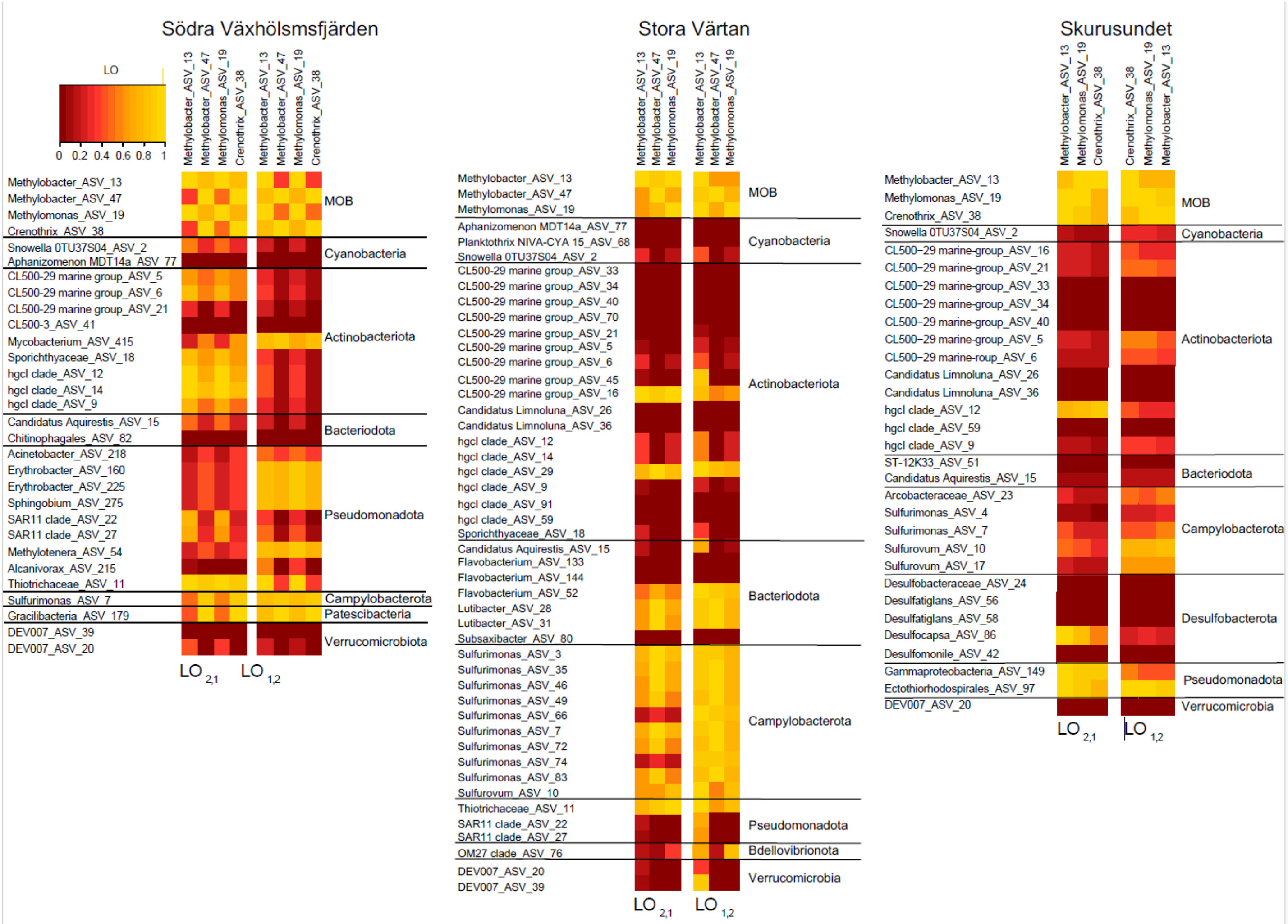

Figure S4: *Niche overlap of MOB ASVs with ASVs with total relative abundance > 1 % and prevalence in > 10 % of all samples per site. Panel LO2,1 reads from left to right (overlap of ASVs with MOB) and LO1,2 reads from top to bottom (overlap of MOB with other ASVs).*

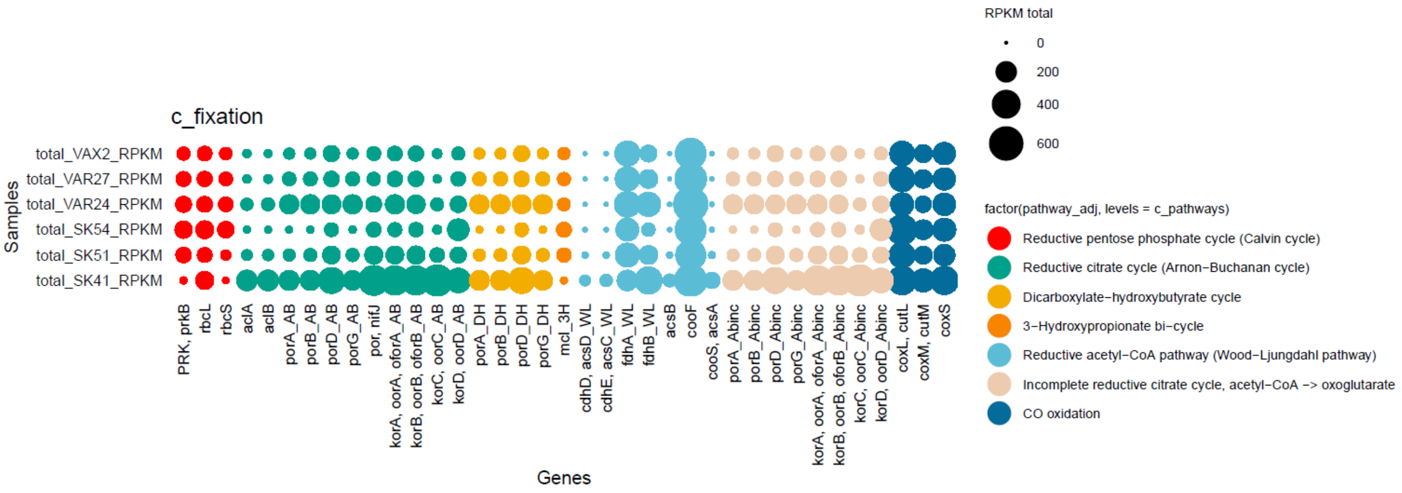

Figure S5: *Overview of carbon fixation genes in the metagenome of samples of different water depths in VAX2 (18m; transition), VAR27 (16m; transition), VAR24 (19m; bottom), SK54(6m; top), SK51 (16m; transition) and SK41 (19m; bottom). Gene read counts of high-quality MAGs were normalised for depth and gene length per sample (RPKM total) and are illustrated as bubble size. Genes were grouped based on kegg pathways and gene names were adapted for genes in multiple pathways (see supplementary data "supplementary_data_3.xlsx").*

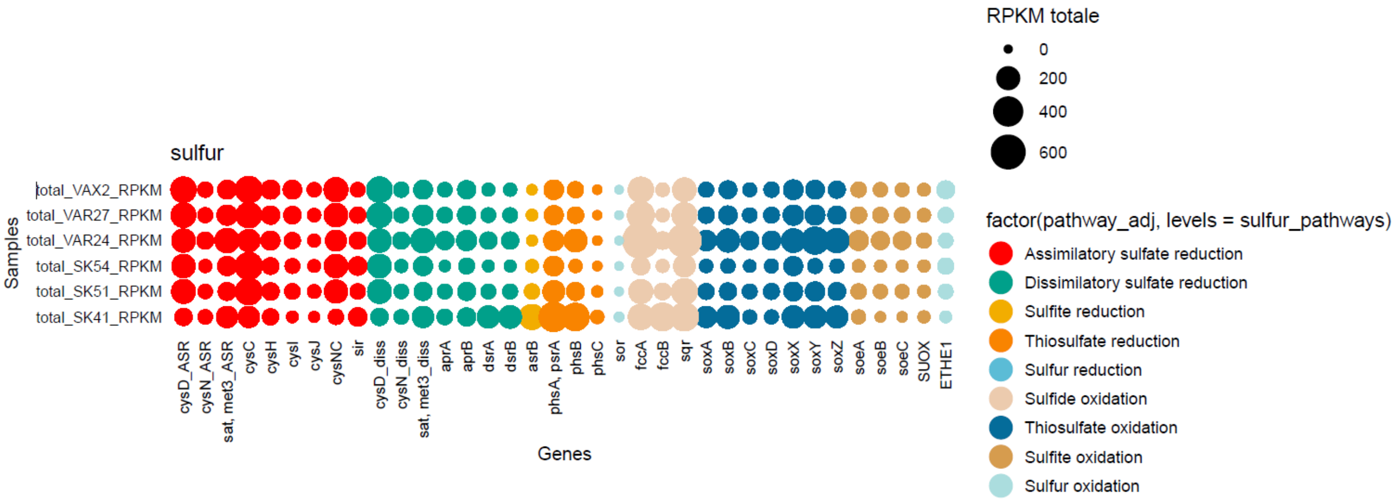

Figure S6: *Overview of sulfur-cycling genes in the metagenome of samples of different water depths in VAX2 (18m; transition), VAR27 (16m; transition), VAR24 (19m; bottom), SK54(6m; top), SK51 (16m; transition) and SK41 (19m; bottom). Gene read counts of high-quality MAGs were normalised for depth and gene length per sample (RPKM total) and are illustrated as bubble size. Genes were grouped based on kegg pathways and gene names were adapted for genes in multiple pathways (see supplementary data "supplementary_data_3.xlsx").*

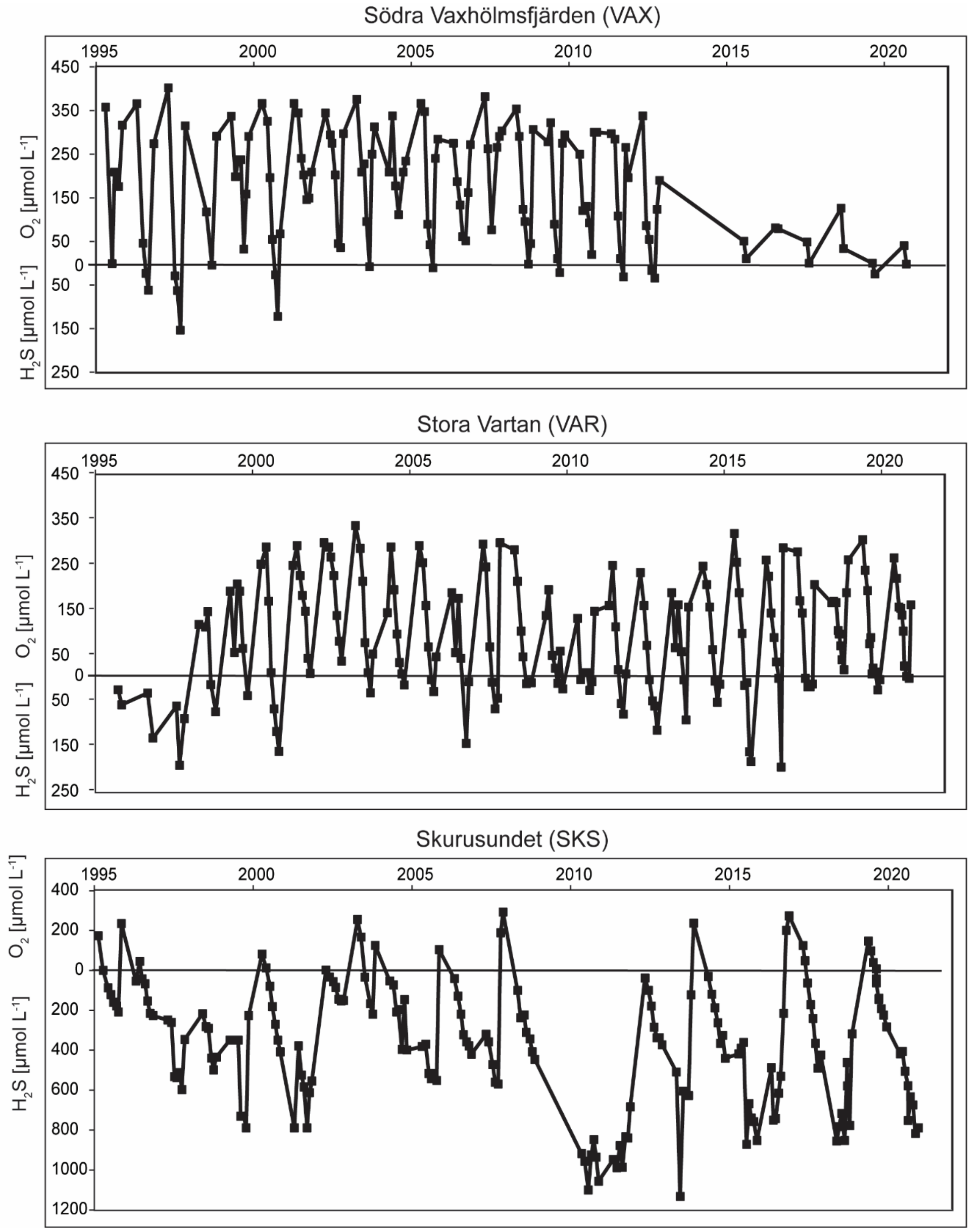

Figure S7: *Monitoring data on bottom water O_2 and H_2S (inverted) concentrations for Södra Växholmsfjärden (VAX), Stora Värtan (VAR) and Skurusundet (SKS) based on Swedish Meteorological and Hydrological Institute; SharkWeb (smhi.se) from 1995 to 2020. See [1] for more information).*

Supplementary tables

Table S1: *Mean methane fluxes and surface water concentrations. Windspeed and temperature were recorded at the time of measuring. Mean and standard deviations of the methane fluxes were calculated from the linear increase of three deployments.*

Site	CH_4 flux $_{water-air}$ [mmol m^{-2} d^{-1}]	std dev	windspeed [m s^{-1}]	temperature [C]	CH_4 SW [nmol L^{-1}]
st 1 (VAX)	0.19	0.011	9.5	4.5	166
st 2 (VAR)	0.13	0.092	3.5	12	112
st 3 (SK)	1.68	0.016	7.5	11	261

Table S2: Overview of depths and number of samples used for the different water layers.

station	water layer	depth [m]	$n_{samples}$
SKS	top	1 - 6	6
SKS	transition	7 - 13	7
SKS	bottom	14 - 24	10
VAR	top	1 - 16	8
VAR	transition	17 - 22	6
VAR	bottom	23 - 27	5
VAX	top	1 - 14	8
VAX	transition	15 - 22	7

Table S3: *Differential abundance analysis between water layers calculated with run_aldex(). Only taxa with a higher abundance than 0.05 % were compared with a t-test. Only differentially abundant taxa with a HB corrected p-value (padj) higher than 0.05 are listed below. Asterix indicate levels of significance (* <0.05, ** < 0.01 and *** <0.001). Number of samples for each tested groups are indicated as $n_{group1,2}$.*

station	group1	group2	Phylum	enrich_group	effect size	pvalue	padj	significance level	n_{group1}	n_{group2}
SKS	top	transition	Actinobacteriota	middle	2,04	0,000000	0,000000	***	6	7
SKS	top	transition	Campylobacterota	top	-2,12	0,001357	0,004070	**	6	7
SKS	top	transition	Cyanobacteria	middle	1,97	0,001621	0,003251	**	6	7
SKS	top	transition	Proteobacteria	top	-2,20	0,001166	0,003961	**	6	7
SKS	top	transition	Verrucomicrobiota	middle	2,15	0,000974	0,002185	**	6	7
SKS	top	bottom	Actinobacteriota	bottom	5,08	0,000000	0,000000	***	6	10
SKS	top	bottom	Bacteroidota	top	-3,90	0,000250	0,000416	***	6	10
SKS	top	bottom	Campylobacterota	top	-3,03	0,000250	0,000416	***	6	10
SKS	top	bottom	Cyanobacteria	bottom	8,27	0,000000	0,000000	***	6	10
SKS	top	bottom	Desulfobacterota	top	-4,47	0,000250	0,000416	***	6	10
SKS	transition	bottom	Actinobacteriota	bottom	1,67	0,000117	0,000347	***	7	10
SKS	transition	bottom	Bacteroidota	middle	-1,37	0,000371	0,001130	**	7	10
SKS	transition	bottom	Cyanobacteria	bottom	1,33	0,000016	0,000063	***	7	10
SKS	transition	bottom	Desulfobacterota	middle	-1,99	0,000227	0,001097	**	7	10
SKS	transition	bottom	Proteobacteria	bottom	1,45	0,001401	0,002803	**	7	10
VAR	top	transition	Actinobacteriota	middle	1,46	0,001519	0,003039	**	8	6
VAR	top	transition	Campylobacterota	top	-2,20	0,000666	0,003996	**	8	6
VAR	top	transition	Cyanobacteria	middle	2,14	0,000000	0,000000	***	8	6
VAR	top	transition	Proteobacteria	top	-2,50	0,001342	0,004027	**	8	6
VAR	top	transition	Verrucomicrobiota	middle	2,12	0,000146	0,000432	***	8	6
VAR	top	bottom	Actinobacteriota	bottom	1,48	0,001962	0,003923	**	8	5
VAR	top	bottom	Bacteroidota	top	-4,42	0,004329	0,008658	**	8	5
VAR	top	bottom	Campylobacterota	top	-2,84	0,004329	0,008658	**	8	5
VAR	top	bottom	Cyanobacteria	bottom	4,17	0,000000	0,000000	***	8	5
VAR	top	bottom	Proteobacteria	top	-3,99	0,004329	0,008658	**	8	5
VAR	top	bottom	Verrucomicrobiota	bottom	5,58	0,000000	0,000000	***	8	5
VAX	top	transition	Actinobacteriota	bottom	1,74	0,000311	0,001088	**	8	7
VAX	top	transition	Bacteroidota	bottom	2,35	0,000311	0,001088	**	8	7
VAX	top	transition	Campylobacterota	top	-1,74	0,000000	0,000000	***	8	7
VAX	top	transition	Cyanobacteria	bottom	1,54	0,002154	0,003093	**	8	7
VAX	top	transition	Planctomycetota	bottom	1,39	0,002159	0,003100	**	8	7
VAX	top	transition	Verrucomicrobiota	bottom	1,47	0,000966	0,002150	**	8	7

Table S4: *Centroids MDS1 and MDS2, variation explained by the model of multiple regression (r^2) and the significance of the multiple regression with 999 permutations (p-values) for each computed environmental vector. Asterix indicate levels of significance (* <0.05, >0;05 n.s.). H_2S concentrations in VAX were below detection limit and therefore nor applicable (n.a.).*

Södra Växholmsfjärden

Env. Factor	MDS1	MDS2	r^2	p-value	signif. level
O_2	-0.61	-0.73	0.91	0.001	*
NH_4	0.68	0.62	0.85	0.001	*
depth	0.42	0.81	0.84	0.001	*
salinity	0.23	0.72	0.57	0.007	*
CH_4	0.16	0.64	0.44	0.023	*
temperature	-0.14	0.48	0.25	0.182	n.s.
H_2S	n.a.	n.a.	n.a.	n.a.	n.a.

Stora Värtan

Env. Factor	MDS1	MDS2	r^2	p-value	signif. level
salinity	-0.99	-0.07	0.98	0.001	*
temperature	0.90	-0.38	0.95	0.001	*
O_2	0.93	0.25	0.92	0.001	*
depth	-0.72	-0.62	0.91	0.001	*
CH_4	-0.81	0.28	0.74	0.001	*
H_2S	-0.78	0.16	0.63	0.001	*
NH_4	-0.74	0.28	0.62	0.001	*

Skurusundet

Env. Factor	MDS1	MDS2	r^2	p-value	signif. level
temperature	0.86	0.43	0.93	0.001	*
NH_4	-0.78	-0.56	0.91	0.001	*
CH_4	-0.55	-0.77	0.91	0.001	*
depth	-0.61	-0.73	0.90	0.001	*
H_2S	-0.56	-0.74	0.86	0.001	*
O_2	0.57	-0.62	0.71	0.001	*
salinity	-0.71	0.11	0.52	0.002	*

Table S5: *Within to between cluster ratios of the different sites and water layers.*

Site	water layer	ratio
VAX	anoxic	0.309
VAX	oxic	0.190
VAR	anoxic	0.167
VAR	hypoxic	0.006
VAR	oxic	0.045
SKS	anoxic	0.000
SKS	hypoxic	0.192
SKS	oxic	0.360

Supplementary methods

Section S1.1. Detailed description of CO_2 concentration calculations

The concentrations in the subsamples (c_a) were calculated with Henry's law. Measured headspace concentrations (c_g) were multiplied by Henry solubility coefficient H^{cc} [2]:

$$c_a = H^{cc} c_g \qquad [1]$$

With the Henry solubility coefficient is defined as follows: $H^{cc} = H^{cp}RT = \beta \frac{1}{RT^{STP}} RT$ [2]

H^{cp}: Henry solubility coefficient (defined as c_a/p)

R: ideal gas constant (8.314 J mol^{-1} K^{-1})

T: temperature (294.14 K)

T^{STP}: the standard temperature for Bunsen coefficient (273.15 K)

β: Bunsen coefficient (including salinity and temperature)

We accounted for the changes in solubility due to salinity and temperature in the calculation of the Bunsen coefficients (Weiss 1970):

$$ln\beta = A_1 + A_2\left(\frac{100}{T}\right) + A_3 ln\left(\frac{T}{100}\right) + S\left[B_1 + B_2\left(\frac{T}{100}\right) + B_3\left(\frac{T}{100}\right)^2\right] \qquad [3]$$

$A_{1\text{-}3}$, $B_{1\text{-}3}$: Bunsen constants, specific for gas

T: Temperature (294.15 K)

S: Salinity (5 ‰)

The Bunsen coefficient for CH_4, CO_2 and O_2 was calculated according to the specific constants for each gas [3–5].

Section 1.2. A detailed description of 16S rRNA amplicon sequencing analysis

Following the DADA2 pipeline [6], obtained raw reads were trimmed (nt 270 forward nt 260 reverse), low-quality reads were removed, and sequences were dereplicated after error 252 models. Then Amplicon sequence variants (ASVs) were inferred, forward and reverse reads were merged, chimaeras removed and taxonomy assigned based on the 254 Silva non-redundant train set v138 downloaded from https://zenodo.org/record/3731176#.XoV8D4gzZaQ. Relative abundances of bacteria and archaea were calculated and illustrated as depth profiles with *phyloseq* and *ggplot2*. Alpha diversity (Chao1 and Shannon) was calculated on untransformed ASV counts per sample with the plot_richness() of the *phyloseq*, illustrated as a boxplot and compared by the Wilcox test (stat_compare_means() *ggpubr*). Beta diversity was depicted in an NMDS plot (metaMDS() *vegan*) and bray-curtis distance was calculated on rarefied data using avgdist() function to the lowest total reads count per sample. Correlation of dispersal and chemical parameters was performed with env_fit() *vegan*. The phylogeny of the retrieved methanotrophic ASVs was performed with the MEGA 11 software [7]. As out-group, reference sequences from representatives of γ-MOB, α-MOB, *Nitrosococcus*, and *Sulfurimonas* were used. The Neigbour-joining tree was computed with 1000 bootstrap iterations. Raw reads of the 16S amplicon sequencing data can be accessed on the National Center for Biotechnology Information (NCBI) database under the accession number PRJNA1126564.

Section 1.3. A detailed description of metagenome sequencing analysis

Metagenomic raw reads were assessed and processed by an adapted in-house pipeline 'binmate' as described in In 't Zandt *et al.*, 2019. Sequencing reads were assessed and pre-processed using FASTQC (v0.11.9) and BBDuk (v37.76). BBDuk was employed for adapter trimming (using the supplied adapters.fa reference; k=23, mink=11, hdist=1, tpe, dbo) and quality trimming (qtrim=lr, trimq=15, ftm=5). Subsequently, Tadpole (v39.06) was used to correct sequencing errors (mode=correct, k=50), and BBnorm (v37.76) to normalized read coverage (target=30, min=2). The reads were co-assembled using metaSPAdes [9] with a range of k-mer sizes (21, 33, 55, 77, 99, 121). Contigs exceeding 2,500 bp were binned using four distinct algorithms: CONCOCT [10], MaxBin2 [11], MetaBAT2 [12], and SemiBin2 [13]. Consensus binning was subsequently performed using DAS Tool [14]. Bin quality was assessed with CheckM [14], with high-quality metagenome-assembled genomes (MAGs) defined as those exhibiting greater than 70% completeness and less than 10% contamination. Taxonomic classification of the bins was achieved using GTBD-Tk [15]. To confirm the phylogeny of the three retrieved MOB MAGS, 231 reference genomes belonging to the *Methylomonadaceae* family were recovered from NCBI (suppmenentary_data_4). In total, 43 conserved genes were found among genoms by CheckM v1.2.2 [16] on lineage_wf mode, which were translated translated using Prodigal [17] with the default parameters and concatenated into one fasta file. The concatenated sequences were aligned using MAFFT v7.45740 [18]. Alignment regions with > 90% gaps were removed with Clipkit [19]. The was then computed using IQTree v2.0.3 [20] using 1000 bootstrapping replicates, and Bayesian information criterion (BIC) for the best model selection.(LG+I+G). Finally, the tree was illustrated with itol iTOL v6 (https://itol.embl.de).

Supplementary results

Section 2.1. Co-occurrence networks

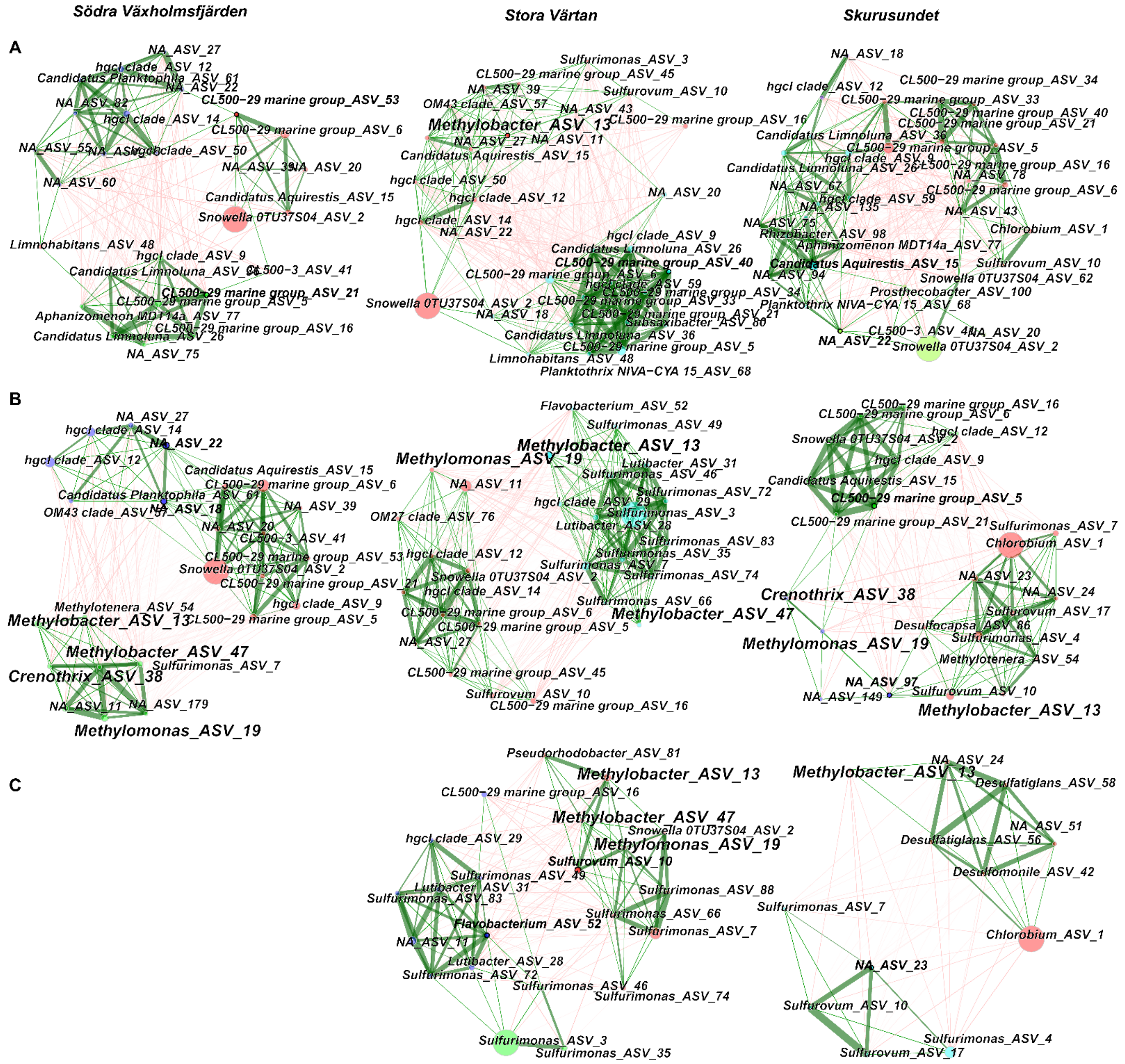

Figure S8 *Co-occurrence networks based on pearson correlation for* **A** *Södra Växhölmsfjärden (VAX),* **B** *Stora Värtan (VAR) and* **C** *Skurusundet (SKS). Edges are displayed as green lines for positive correlations and red lines negative correlations. Strong positive correlations are emphasized with line width. Node colours indicate clusters and node size indicates abundance. Hubs based on betweenness are indicated with black node borders and bold text. Methane oxidizing bacteria are highlighted with a bigger font.*

The Pearson correlation-based co-occurrence networks of samples grouped by site and water layer, illustrated the differences in community connectivity and importance of certain taxa (Fig 3). Most notably, in the stratified sites VAR and SKS, the overall node degree was higher at the top layer compared to the bottom water layers. In the bottom and transition water layers of VAR, MOB ASVs were predominantly connected to sulfur-oxidizing bacteria. Most notably, in the stratified sites VAR and SKS, the overall node degree was

higher at the top layer compared to the bottom water layers. In the bottom and transition water layers of VAR, MOB ASVs were predominantly connected to sulfur-oxidizing bacteria. However, *Methylobacter_ASV_13* clustered with the dominant Actinobacterota in the top layer as did *Meyhlomonas_ASV_19* in the transition layer. In SKS*, Methylomonas_ASV_19* and *Crenothrix_ASV_38* in the transition zone built a peripheral cluster together with *Pseudomonadota_ASV_149* and *Ecrothiorhodospirales_ASV_97* and connected the Cyanobacteria/Actinobacteriota cluster with the sulfur-cycling cluster. Notably, Campylobacterota and Desulfobacterota were separated into two unconnected clusters in the bottom layer. MOB *Methylobacter_ASV_13* (< 2 %) clustered with members of Desulfobacterota but not with Campylobacterota as in VAR. In VAX, all MOB ASVs clustered with Sulfurimonas, Thiothrichaceae and Methylotenera and the connectedness did not differ much.

Section 2.2 Phylogeny of MOB ASVs

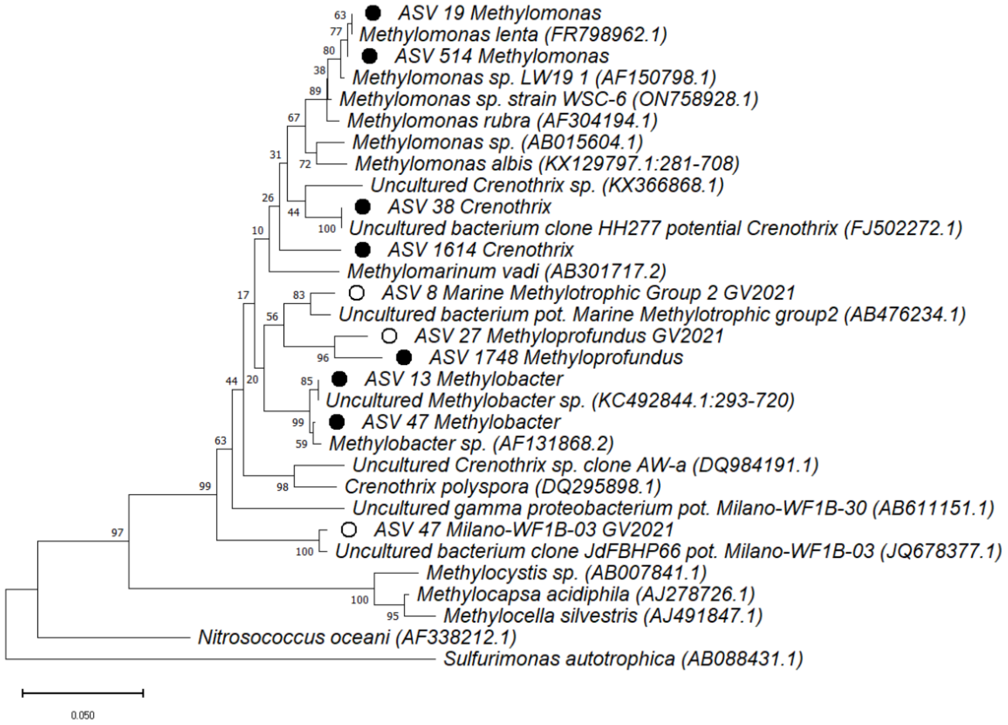

Figure S9: *Phylogeny of Methanotrophic ASVs retrieved from 16S amplicon sequencing (indicated with full circles)—neighbour-joining tree with sequences from other γ-MOB retrieved from the ENA database. Representatives were chosen from Methylobacter, Methylomonas and Crenothrix for comparison. Methyloprofundus, Marine methylotrophic group 2 and Milano -WB1F-03 sequences were added as additional representatives of the Methylomonadaceae family. Methylocella, Methylocapsa and Methylocystis sequences were used to represent the α-MOB. Nitrosococcus and Sulfurimonas were used as out-groups. Alignment and tree computation were performed with the MEGA11 software (Tamura et al., 2021). Sequences were aligned with the ClustalW function, and the Neighbour-joining tree was computed with 1000 bootstrap iterations (values at branching points [%]).*

Phylogenetic analysis showed that the amplicons assigned to *Methylomonas* and *Methylobacter* grouped with several different species (Fig. S6). *Methylomonas_ASV_19* and *Methylomonas_ASV_514* grouped with *Methylomonas lenta, a species* originally isolated from the denitrification tank of a wastewater treatment plant [21]. The *Methylobacter* ASVs were related to different uncultured *Methylobacter* sequences from the

redoxcline of the Baltic Sea [22] and swamp soil [23]. None of the ASVs assigned to *Crenothrix* grouped with the *Crenothrix polyspora* culture sequences. *Crenothrix_ASV_38* clustered with an *uncultured bacterium clone HH277* from a chemocline sample in Alpine Lake Cadagno [24] both of which did not score an identity higher than 96.73 % (*Methylomonas sp.* KX129797.1) with any taxonomically assigned sequence in the NCBI database. Moreover, *Crenothrix_ASV_13* branched away from uncultured *Crenothrix sp.* (KX366868.1) with a low bootstrap value and was separated from the other *Crenothrix_ASV_1614*.

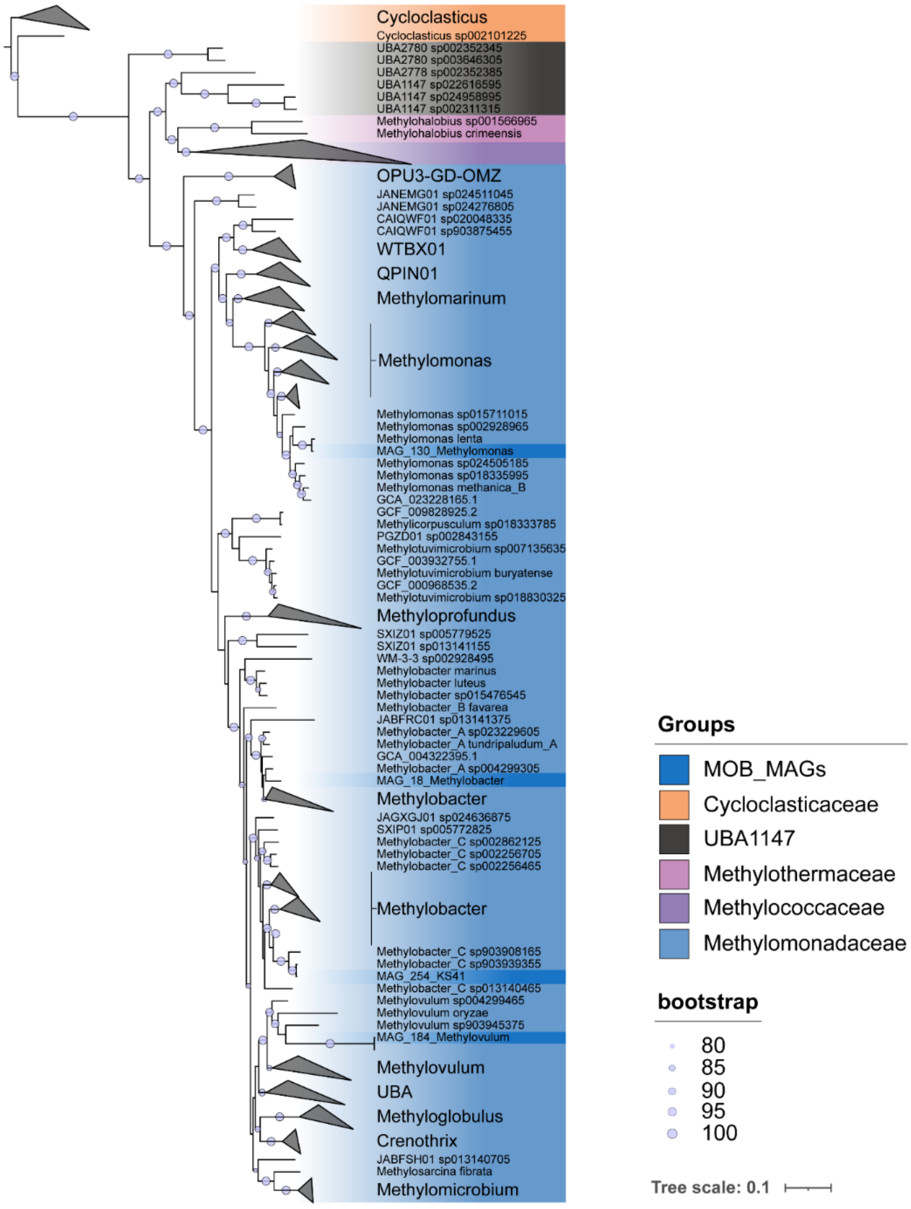

References

1. Żygadłowska OM, Roth F, van Helmond NAGM *et al.* Eutrophication and Deoxygenation Drive High Methane Emissions from a Brackish Coastal System. *Environ Sci Technol* 2024, DOI: 10.1021/acs.est.4c00702.

2. Sander R. Compilation of Henry's law constants (version 4.0) for water as solvent. *Atmos Chem Phys* 2015;**15**:4399–981.

3. Weiss RF. The solubility of nitrogen, oxygen and argon in water and seawater. *Deep Sea Research and Oceanographic Abstracts* 1970;**17**:721–35.

4. Weiss RF. Carbon dioxide in water and seawater: the solubility of a non-ideal gas. *Marine Chemistry* 1974;**2**:203–15.

5. Yamamoto S, Alcauskas JB, Crozier TE. Solubility of methane in distilled water and seawater. *Journal of Chemical & Engineering Data* 1976;**21**:78–80.

6. Callahan BJ, McMurdie PJ, Holmes SP. Exact sequence variants should replace operational taxonomic units in marker-gene data analysis. *The ISME Journal* 2017;**11**:2639–43.

7. Tamura K, Stecher G, Kumar S. MEGA11: Molecular Evolutionary Genetics Analysis Version 11. *Molecular Biology and Evolution* 2021;**38**:3022–7.

8. In 't Zandt, Kip Nardy, Frank Jeroen *et al.* High-Level Abundances of Methanobacteriales and Syntrophobacterales May Help To Prevent Corrosion of Metal Sheet Piles. *Applied and Environmental Microbiology* 2019;**85**:e01369-19.

9. Nurk S, Meleshko D, Korobeynikov A *et al.* metaSPAdes: a new versatile metagenomic assembler. *Genome research* 2017;**27**:824–34.

10. Alneberg J, Bjarnason BS, De Bruijn I *et al.* Binning metagenomic contigs by coverage and composition. *Nature methods* 2014;**11**:1144–6.

11. Wu Y-W, Simmons BA, Singer SW. MaxBin 2.0: an automated binning algorithm to recover genomes from multiple metagenomic datasets. *Bioinformatics* 2016;**32**:605–7.

12. Kang DD, Li F, Kirton E *et al.* MetaBAT 2: an adaptive binning algorithm for robust and efficient genome reconstruction from metagenome assemblies. *PeerJ* 2019;**7**:e7359.

13. Pan S, Zhao X-M, Coelho LP. SemiBin2: self-supervised contrastive learning leads to better MAGs for short-and long-read sequencing. *Bioinformatics* 2023;**39**:i21–9.

14. Sieber CM, Probst AJ, Sharrar A *et al.* Recovery of genomes from metagenomes via a dereplication, aggregation and scoring strategy. *Nature microbiology* 2018;**3**:836–43.

15. Chaumeil P-A, Mussig AJ, Hugenholtz P *et al.* GTDB-Tk v2: memory friendly classification with the genome taxonomy database. *Bioinformatics* 2022;**38**:5315–6.

16. Parks DH, Imelfort M, Skennerton CT *et al.* CheckM: assessing the quality of microbial genomes recovered from isolates, single cells, and metagenomes. *Genome research* 2015;**25**:1043–55.

17. Hyatt D, Chen G-L, LoCascio PF *et al.* Prodigal: prokaryotic gene recognition and translation initiation site identification. *BMC bioinformatics* 2010;**11**:1–11.

18. Katoh K, Standley DM. MAFFT multiple sequence alignment software version 7: improvements in performance and usability. *Molecular biology and evolution* 2013;**30**:772–80.

19. Steenwyk JL, Buida III TJ, Li Y *et al.* ClipKIT: a multiple sequence alignment trimming software for accurate phylogenomic inference. *PLoS biology* 2020;**18**:e3001007.

20. Nguyen L-T, Schmidt HA, Von Haeseler A *et al.* IQ-TREE: a fast and effective stochastic algorithm for estimating maximum-likelihood phylogenies. *Molecular biology and evolution* 2015;**32**:268–74.

21. Hoefman S, Heylen K, De Vos P. Methylomonas lenta sp. nov., a methanotroph isolated from manure and a denitrification tank. *International Journal of Systematic and Evolutionary Microbiology* 2014;**64**:1210–7.

22. Glaubitz Sabine, Kießlich Katrin, Meeske Christian *et al.* SUP05 Dominates the Gammaproteobacterial Sulfur Oxidizer Assemblages in Pelagic Redoxclines of the Central Baltic and Black Seas. *Applied and Environmental Microbiology* 2013;**79**:2767–76.

23. Ren T, Roy R, Knowles R. Production and consumption of nitric oxide by three methanotrophic bacteria. *Appl Environ Microbiol* 2000;**66**:3891–7.

24. Halm H, Musat N, Lam P *et al.* Co-occurrence of denitrification and nitrogen fixation in a meromictic lake, Lake Cadagno (Switzerland). *Environmental Microbiology* 2009;**11**:1945–58.
